# Autophagy and aging: Maintaining the proteome through exercise and caloric restriction

**DOI:** 10.1111/acel.12876

**Published:** 2018-11-15

**Authors:** Kurt A. Escobar, Nathan H. Cole, Christine M. Mermier, Trisha A. VanDusseldorp

**Affiliations:** ^1^ Department of Kinesiology California State University, Long Beach Long Beach California; ^2^ Department of Health, Exercise, & Sports Sciences University of New Mexico Albuquerque New Mexico; ^3^ Department of Exercise Science & Sports Management Kennesaw State University Kennesaw Georgia

**Keywords:** aging, autophagy, caloric restriction, exercise, mTOR, physical activity

## Abstract

Accumulation of dysfunctional and damaged cellular proteins and organelles occurs during aging, resulting in a disruption of cellular homeostasis and progressive degeneration and increases the risk of cell death. Moderating the accrual of these defunct components is likely a key in the promotion of longevity. While exercise is known to promote healthy aging and mitigate age‐related pathologies, the molecular underpinnings of this phenomenon remain largely unclear. However, recent evidences suggest that exercise modulates the proteome. Similarly, caloric restriction (CR), a known promoter of lifespan, is understood to augment intracellular protein quality. Autophagy is an evolutionary conserved recycling pathway responsible for the degradation, then turnover of cellular proteins and organelles. This housekeeping system has been reliably linked to the aging process. Moreover, autophagic activity declines during aging. The target of rapamycin complex 1 (TORC1), a central kinase involved in protein translation, is a negative regulator of autophagy, and inhibition of TORC1 enhances lifespan. Inhibition of TORC1 may reduce the production of cellular proteins which may otherwise contribute to the deleterious accumulation observed in aging. TORC1 may also exert its effects in an autophagy‐dependent manner. Exercise and CR result in a concomitant downregulation of TORC1 activity and upregulation of autophagy in a number of tissues. Moreover, exercise‐induced TORC1 and autophagy signaling share common pathways with that of CR. Therefore, the longevity effects of exercise and CR may stem from the maintenance of the proteome by balancing the synthesis and recycling of intracellular proteins and thus may represent practical means to promote longevity.

## 1 INTRODUCTION

Aging is a biological phenomenon characterized at the cellular level by a progressive accumulation of dysfunctional proteins and damaged organelles. Accrual and aggregation of these defunct components result in disruption of cellular homeostasis, progressive degeneration, and increases the risk of cell death (Lopez‐Otin, Blasco, Partridge, Serrano, & Kroemer, 2013). Accordingly, it has been proposed that escalating malfunction in the regulatory processes required for the maintenance, repair, and turnover of defective protein structures and organelles is likely to represent a primary cause of the cumulative cellular disorganization associated with aging (Demontis & Perrimon, 2010; Madeo, Zimmermann, Maiuri, & Kroemer, 2015). Autophagy is an evolutionary conserved cellular housekeeping pathway responsible for the degradation of misfolded proteins and exhausted organelles and has been increasingly demonstrated to play a major role in maintaining cellular homeostasis and influencing lifespan and longevity (Filfan et al., 2017; Madeo et al., 2015; Madeo, Tavernarakis, & Kroemer, 2010). Compromised autophagic capability facilitates reduced lifespan and precipitates premature aging in numerous model species (Alvers et al., 2009; Hars et al., 2007; Juhasz, Erdi, Sass, & Neufeld, 2007; Kang, You, & Avery, 2007; Toth et al., 2008), while enhanced autophagy has been shown to promote longevity (Demontis & Perrimon, 2010; Eisenberg et al., 2009; Pyo et al., 2013; Simonsen et al., 2008). Moreover, autophagic activity appears to decline naturally with age (Cuervo & Macian, 2014; Donati, Recchia, Cavallini, & Bergamini, 2008; Mejias‐Pena et al., 2016), thus progressively challenging proteostasis and contributing to the accumulation of inutile cellular components often associated with aging (Madeo et al., 2015).

Intracellular protein quality concurrently depends upon protein synthesis (Salminen & Kaarniranta, 2009). As such, the degradation of superfluous and dysfunctional cytosolic components, as occurs through autophagy, represents only one aspect of intracellular protein accumulation, which is ultimately balanced by the regulatory elements managing synthesis of new cellular proteins.

The target of rapamycin complex 1 (TORC1; known as mTORC1 in mammalian species) is a central regulatory kinase that regulates cellular growth and protein synthesis. This complex is stimulated by nutrient availability (i.e., amino acids), mechanical stress, and growth factors (i.e., insulin‐like growth factor 1 [IGF‐1]) and is inhibited by nutrient deprivation, energetic stress, and the macrocyclic polyketide rapamycin (Chantranupong et al., 2014; Jung, Ro, Cao, Otto, & Kim, 2010; Meijer, Lorin, Blommaart, & Codogno, 2015). Recently, TORC1 activity has been linked to lifespan and the aging process whereby inhibition of the TORC1 pathway is consistently observed to enhance longevity in animal and cellular models (Lamming, Ye, Sabatini, & Baur, 2013; Pani, 2011; Xu, Cai, & Wei, 2014). While TORC1 moderation of lifespan has been reported in a variety of model organisms, the underlying mechanisms have yet to be cogently elucidated (Kaeberlein, 2013; Xu et al., 2014). However, it has been long understood that TORC1 serves as an inhibitor of autophagy, and thus, it has further been suggested that autophagy represents a key link between TORC1 activity and the aging process (Pani, 2011; Wei, Zhang, & Cai, 2013; Xu et al., 2014). The TORC1 pathway may then dually contribute to the detrimental accumulation of cytosolic proteins observed during aging, acting both through upregulation of protein synthesis and the downregulation of autophagic degradation (Laplante & Sabatini, 2012; Xu et al., 2014).

Caloric restriction (CR) has been shown to be a reliable method of lifespan extension and/or moderator of age‐related disease through modulation of autophagic activity in numerous model species ranging from yeast to humans (Bitto et al., 2016; Morselli et al., 2010; Most, Tosti, Redman, & Fontana, 2016). Similarly, regular exercise has long been known to promote healthy aging and mitigate age‐related disease (Booth, Roberts, & Laye, 2012; Bouzid, Filaire, McCall, & Fabre, 2015; Vina, Rodriguez‐Manas, Salvador‐Pascual, Tarazona‐Santabalbina, & Gomez‐Cabrera, 2016). Though the mechanisms underlying the exercise‐mediated effects on longevity have yet to be fully understood, exercise also influences autophagic and mTORC1 activity in rodent and human models (Halling, Ringholm, Olesen, Prats, & Pilegaard, 2017; He, Bassik, et al., 2012; Jamart, Benoit, et al., 2012; Schwalm et al., 2015). Moreover, CR and exercise exert their effects on autophagy and mTORC1 activity through common pathways in rodent and human models (Egan et al., 2011; Medina et al., 2015; Ng & Tang, 2013; Tam & Siu, 2014; Watson & Baar, 2014). While presently the long‐term effects of chronic exercise on the interplay between these proteostatic systems and longevity have yet to be characterized, the robust effects of exercise on the aging process may in large part mirror those of CR given their shared modulatory roles in autophagy and mTORC1 activity. This review will discuss the current literature relating autophagy and mTORC1 activity to the aging process and highlight evidence of the effects of CR and exercise on these regulatory pathways, as well as the associated implications for healthy human aging.

## 2 HOUSEKEEPING AND AGING: AUTOPHAGY‐MEDIATED EFFECTS

Autophagy is a proteostatic process that has been highly conserved throughout evolution and is present in all known eukaryotic cells, from yeast to humans (Madeo et al., 2015; Most et al., 2016). The umbrella term “autophagy” is often subdivided into three primary pathways, each dependent on lysosomal degradation, which are chaperone‐mediated autophagy, microautophagy, and macroautophagy (Feng, He, Yao, & Klionsky, 2014). Macroautophagy is currently best understood (as well as the primary type of autophagy studied within the context of exercise) (Halling & Pilegaard, 2017; Vainshtein & Hood, 2016) and will serve as the focus in the current discussion, being referred to as autophagy hereafter. This process functions through bulk (Feng et al., 2014) as well as selective degradation (Johansen & Lamark, 2011; Li & Vierstra, 2012) of cellular material including organelles, cytosolic proteins, and protein aggregates; all of which are sequestered by double‐membrane vesicles called autophagosomes and then transported to the lysosome for degradation (Feng et al., 2014). Various proteins, designated as autophagy‐related genes (Atgs), associated with sequestering cytosolic components and autophagosome formation have been identified as crucial to normal autophagic function largely through the study of mutant model organisms deficient in autophagic activity (Feng et al., 2014). Notably, TORC1 negatively regulates autophagy by directly interacting with Atgs, ultimately preventing the formation of the autophagosome (Kim & Guan, 2015; Meijer et al., 2015). More specifically, hyperphosphorylation of Atg13 and Atg1 (known as ULK1 in mammals) by TORC1 prevents the association of these proteins, which is required to initiate autophagosome formation (Meijer et al., 2015). Additionally, TORC1 inhibits autophagy at the transcriptional level (Martina, Chen, Gucek, & Puertollano, 2012). Transcription factor EB (TFEB), the primary regulator of cellular recycling that coordinates the expression of lysosomal and autophagic genes via the CLEAR (coordinated lysosomal expression and regulation) network (Sardiello et al., 2009; Settembre et al., 2011), is phosphorylated by TORC1 on the lysosomal membrane, thus preventing its translocation to the nucleus and subsequent transcription of Atgs (Martina et al., 2012).

An age‐related decline in overall proteolytic activity has been observed in a broad range of organisms, and the progressive accumulation of damaged proteins with age has been extensively documented (Demontis & Perrimon, 2010; Liang & Jung, 2010; Martinez‐Lopez, Athonvarangkul, & Singh, 2015; Rajawat & Bossis, 2008). Moreover, a natural decline in autophagic function has been reported in several specific organs and tissues with advancing age and has been observed across a number of model species, including mammals (Donati et al., 2008; Martinez‐Lopez et al., 2015; Mejias‐Pena et al., 2016; Phadwal et al., 2012). The degenerative loss of autophagic activity in aged cells is likely to increasingly constrain the ability of the cell to sustain a healthy proteome and organelle population, contributing to a progressive loss of cellular function, and eventually precipitating cell death (Cuervo & Macian, 2014; Rubinsztein, Marino, & Kroemer, 2011). Though the mechanisms underlying the escalating impairment of autophagic function in aging cells remain poorly understood, decreased Atg expression at the mRNA and protein level has been implicated as a contributing factor (Carames, Taniguchi, Otsuki, Blanco, & Lotz, 2010; Lipinski et al., 2010; Rubinsztein et al., 2011). It has also been reported that ancillary proteins necessary for the induction of autophagy, such as Sirtuin 1 (SIRT1), display a similarly reduced expression in aged cells, concomitant with diminished autophagy (de Kreutzenberg et al., 2010; Rubinsztein et al., 2011). At present, it remains unclear whether these decrements in Atgs and/or upstream signaling targets are the primary source of age‐dependent autophagic malfunction (Martinez‐Lopez et al., 2015; Rubinsztein et al., 2011), as it has also been suggested that the decline in basal autophagy may be at least partially mediated by excess TORC1 activity (Lee et al., 2010; Pani, 2011; Xu et al., 2014).

To date, it has been well documented that inhibition of autophagy results in premature aging across a variety of species (Rubinsztein et al., 2011). Loss‐of‐function mutations in select Atg proteins (Atg1, Atg7, Atg18, and beclin‐1) have been directly demonstrated to decrease lifespan in the nematode *Caenorhabditis elegans* (*C. elegans*) (Toth et al., 2008). Similarly, silencing the expression of Atg1, an essential protein of autophagosome formation, was also observed to significantly reduce lifespan in the fruit fly *Drosophila melanogaster* (*D. melanogaster*) (Lee et al., 2010). In mice, knockout of Atg proteins engenders age‐associated defects, including accumulation of dysfunctional organelles (Hartleben et al., 2010; Komatsu et al., 2005; Masiero et al., 2009), abnormal protein aggregation (Liang & Jung, 2010; Liang, Wang, Peng, Gan, & Guan, 2010; Wu et al., 2009), disorganized mitochondria (Komatsu et al., 2005; Masiero et al., 2009), and endoplasmic stress (Hartleben et al., 2010).

Moreover, an accumulating body of evidence has suggested that lifespan extension can result from a maintained autophagic function with experimentally enhanced autophagy shown to delay the aging phenotype and extend longevity (Martinez‐Lopez et al., 2015; Rajawat, Hilioti, & Bossis, 2009; Rubinsztein et al., 2011). It has been observed that various interventions leading to an upregulation of autophagic activity can extend longevity in *C. elegans* (Hansen et al., 2008; Melendez et al., 2003), as well as in the yeast *Saccharomyces cerevisiae* (*S. cerevisiae*) (Eisenberg et al., 2009), while also promoting longevity in individual cells and tissues (Demontis & Perrimon, 2010; Donati, Taddei, Cavallini, & Bergamini, 2006). Thus, augmenting autophagic function may represent a therapeutic target in promoting longevity in humans.

## 3 TOR AND AGING

Target of rapamycin complex 1 (mTORC1 in mammals) is one of two functionally and compositionally distinct multi‐protein TOR complexes; the second being TOR complex 2 (TORC2). Both complexes are highly conserved in all known eukaryotic cells (Meijer et al., 2015; Xu et al., 2014). TORC1 is a primary mediator of protein synthesis and cell growth, whereas TORC2 remains poorly understood. TORC2 has been suggested to regulate spatial coordination of the cytoskeleton (Sarbassov et al., 2004; Xu et al., 2014), while also being involved in TORC1 activation via the Akt pathway (Jung et al., 2010; Sarbassov et al., 2006; Sarbassov, Guertin, Ali, & Sabatini, 2005). Acute rapamycin treatment strongly inhibits TORC1 activity, but the effects of TORC2 are not fully characterized as rapamycin cannot bind to the fully assembled TORC2 complex (Kaeberlein, 2013; Xu et al., 2014). However, it has been demonstrated that chronic rapamycin treatment can also disrupt TORC2 activity by preventing the formation of the TORC2 complex (Sarbassov et al., 2006). Accordingly, in research involving long‐term rapamycin treatment, especially those related to research on aging, the role of TORC2 remains unclear (Kaeberlein, 2013; Xu et al., 2014).

It has been well established that inhibition of the TORC1 pathway results in extended lifespan and promotes healthy aging in numerous model species (Kaeberlein, 2013; Kapahi et al., 2010; Laplante & Sabatini, 2012; Xu et al., 2014). While TORC1 is also known to act as an inhibitor of autophagy, it has yet to be conclusively established that the lifespan‐extending effects of TORC1 suppression are directly attributable to subsequent increased autophagic activity, the reduction of the synthesis of new cellular proteins, or some combination of the two (Kaeberlein, 2013; Kapahi et al., 2010; Meijer et al., 2015; Pani, 2011; Xu et al., 2014). It may be that continuing protein synthetic activity via TORC1 in postmitotic cells (i.e., mature cells which have entered cell cycle arrest, and no longer replicate) leads to an overload of the mechanisms responsible for cellular degradation, including autophagy, and the accumulation of superfluous cytosolic components. Eventually, an insufficiency of degradation systems in these senescent cells results in protein aggregation and pathological cellular disorganization (Pani, 2011; Xu et al., 2014).

The initial observation of extended lifespan accompanying TORC1 inhibition was made in *C. elegans*, where reducing TORC1 activity increased lifespan more than twofold (Vellai et al., 2003). Similar findings have been reported in numerous species using multiple methods of mTORC1 inactivation (Kapahi et al., 2010; Pani, 2011; Xu et al., 2014). In mice, the direct genetic knockdown of mTORC1 resulted in a 20% lifespan extension and a prominent reduction in age‐associated pathologies (Wu et al., 2013). Administration of rapamycin initiated at 600 days in mice, an age analogous to approximately 50 years in humans, extended lifespan up to 14% in female and 9% in male animals (Harrison et al., 2009). Additionally, 3 months of rapamycin treatment increased life expectancy by up to 60% in middle‐aged mice (Bitto et al., 2016). Downregulation of TORC1 activity in *D. melanogaster* through genetic manipulation of the upstream nutrient‐sensing pathways normally responsible for activating TORC1 also extended lifespan by approximately 15% (Kapahi et al., 2004). In counterpoint, silencing expression of Sestrin, a TORC1 inhibitor, has been shown to instigate numerous age‐related pathologies, which were then prevented by pharmacological inhibition of TORC1 in *D. melanogaster* (Lee et al., 2010).

In addition, manipulation of the TORC1 activator ras homologue in brain (Rheb) (Honjoh, Yamamoto, Uno, & Nishida, 2009), as well as downstream targets of TORC1, such as S6K (a ribosomal kinase involved in translation), and eukaryotic translation initiation factor (known as 4E‐BP1), has also been shown to produce significant lifespan extension in a variety of model organisms (Kapahi et al., 2010; Xu et al., 2014). With regard to the downstream targets of TORC1 involved in gene translation, deletion of the gene encoding for the homologue of human S6K1 in yeast (Sch9) has been reported to produce up to a 90% increase in lifespan (Fabrizio, Pozza, Pletcher, Gendron, & Longo, 2001). Similarly, mRNA knockdown of the S6K1 homologue in *C. elegans* has been reported to extend longevity by a mean of 22% (Pan et al., 2007). This effect was potentiated to 46% by simultaneous suppression of the eIF4G homologue, which is another key initiator of gene translation known to be positively regulated by TORC1 (Pan et al., 2007). In mice, the knockout of S6K1 has also been shown to extend mean lifespan by approximately 19% (Selman et al., 2009). Similarly, overexpression of 4E‐BP1 in *D. melanogaster*, which is negatively regulated by TORC1 and inhibits translation initiation by suppressing eIF4G, increased lifespan by 11% and 22% in males and females, respectively (Zid et al., 2009). Similarly, reduced cytosolic protein synthesis has been shown to suppress age‐associated mitochondrial degeneration in yeast (Wang, Zuo, Kucejova, & Chen, 2008).

This evidence suggests that normal levels of autophagy may be sufficient to maintain cytosolic proteostasis if the rate of protein and organelle synthesis is reduced; however, it may also be possible that the reduced levels of autophagy observed in older cells may not be linked to aging, but simply offer an indirect reflection of excess TORC1 activity (Pani, 2011). At present, it remains unclear whether the lifespan‐extending effects of TORC1 pathway inhibition are primarily attributable to reductions in protein synthetic activity, to the removal of autophagy inhibition, or to a combination of these effects (Kaeberlein, 2013; Rubinsztein et al., 2011). However, it has been observed that knockdown of Atg genes critical to autophagic function abrogates the life‐extending effects of rapamycin, suggesting autophagy does possess a key role in TORC1‐mediated life extension (Bjedov et al., 2010; Rubinsztein et al., 2011).

## 4 CALORIC RESTRICTION PROMOTES LIFESPAN AND HEALTH IN AGING

**Figure 1:**
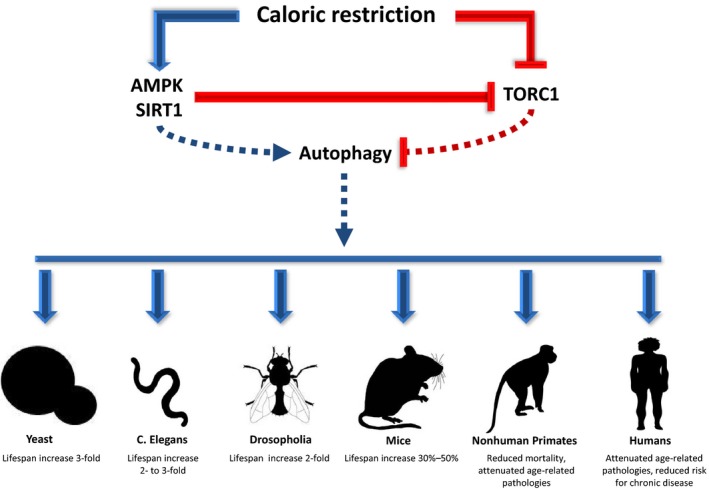
Influence of caloric restriction on life span and age‐related pathologies in various model organisms and potential underlying pathways (represented by dashed lines). Caloric restriction activates 5′ adenosine monophosphate kinase (AMPK) and Sirtuin‐1 and downregulates target of rapamycin complex 1 (TORC1). AMPK and SIRT1 in turn stimulate autophagy and further inhibit TORC1. Blue arrow head and red capped head represent activation and inhibition, respectively

Lifespan extension via autophagy has been closely linked to CR (Bergamini, Cavallini, Donati, & Gori, 2007; Madeo et al., 2015). In this parlance, CR is defined as a sustained decrement in daily energy intake, which yet remains adequate to avoid evoking malnutrition, and typically corresponds to 20%–40% caloric reduction in higher mammals (Bergamini et al., 2007; Mirzaei, Suarez, & Longo, 2014). Caloric restriction has been demonstrated to enhance lifespan and/or reduce many pathological manifestations of aging in a wide range of organisms, from yeast, *S. cerevisiae* and *C. elegans* to rodents and primates, including humans (Figure 1) (Colman et al., 2009; Fontana, Partridge, & Longo, 2010; Madeo et al., 2015; Mirzaei et al., 2014; Rubinsztein et al., 2011; Weindruch, Walford, Fligiel, & Guthrie, 1986), and represents the only known nongenetic intervention to promote these indications in higher organisms (Wang, Liang, & Vanhoutte, 2011). Caloric restriction has been shown to promote health and protect against a number of age‐related pathologies in humans including cancer, type 2 diabetes, cardiovascular disease, nephropathy, and neurodegenerative disease (Cangemi, Friedmann, Holloszy, & Fontana, 2010; Fontana & Klein, 2007; Fontana, Meyer, Klein, & Holloszy, 2004; Meyer et al., 2006; Most et al., 2016; Stein et al., 2012; Yang et al., 2014).

Even modest implementations of CR, such as intermittent fasting protocols, can promote health (Brandhorst et al., 2015; Martin, Mattson, & Maudsley, 2006; Wei et al., 2017; Zuo et al., 2016). Six days of mild CR followed by 1 day of fasting (120 kcal), rendering a weekly CR of 30%, improved body composition, plasma lipids, and adipokines (Kroeger et al., 2012). Additionally, various fasting interventions have demonstrated improvements in symptomology in type 2 diabetes (Barnosky, Hoddy, Unterman, & Varady, 2014). Wei et al. (2017) recently showed reducing energy intake (to 750–1,100 kcal/day) for only five consecutive days per month for 3 months resulted in improvements in body composition, blood pressure, fasting glucose, triglycerides, total and low‐density lipoprotein cholesterol, C‐reactive protein, and IGF‐1. Notably, IGF‐1 is an upstream regulator of TORC1 (Jung et al., 2010).

Indeed, it has been observed that long‐term CR is a strong physiological promoter of autophagy, resulting in an upregulation of a number of autophagy‐related modulators and transcripts (Mercken et al., 2013; Yang et al., 2016). Caloric restriction‐mediated autophagy activity is largely accomplished through activation of the nutrient sensors 5' adenosine monophosphate‐activated protein kinase (AMPK) and SIRT1 (Egan et al., 2011; Meijer et al., 2015; Ng & Tang, 2013). AMPK is a highly conserved kinase that becomes activated during periods of energetic stress, when reductions of ATP precipitates increased intracellular AMP and ADP concentrations, such as during nutrient starvation or exercise (Gwinn et al., 2008; Hawley & Houmard, 2004; Salminen & Kaarniranta, 2012). Moreover, crosstalk between AMPK and SIRT1, an NAD^+^‐dependent protein deacetylase also sensitive to energetic challenges, has been implicated in mediating the aging process (Salminen & Kaarniranta, 2012; Wang et al., 2011). These effectors act to augment the activity of transcriptional factors involved with the expression of several Atgs, including FOXO1 and FOXO3 (Salminen & Kaarniranta, 2012; Vainshtein & Hood, 2016) as well as exert inhibitory effects on TORC1 (Ghosh, McBurney, & Robbins, 2010; Wang et al., 2011). Notably, these signaling targets are involved in the longevity‐promoting effects of metformin (Cabreiro et al., 2013; Mouchiroud, Molin, Dalliere, & Solari, 2010) as well as the acute response to exercise (Hawley, Hargreaves, Joyner, & Zierath, 2014).

Additionally, TFEB, a transcription factor involved in coordinating the expression of lysosomal and autophagic genes, has been shown to be activated during energy deprivation (Medina et al., 2015). At energy balance, TFEB is phosphorylated by mTORC1 on the lysosomal membrane preventing its translocation to the nucleus. During starvation, mTORC1 disassociates from the lysosome, releasing TFEB. At the same time, Ca^++^ is released from the lysosome into the cytosol, activating calcineurin which dephosphorylates TFEB and promotes its translocation to the nucleus where it initiates the transcription of a number of Atgs and proteins (Palmieri et al., 2011; Settembre et al., 2011). Interestingly, the response is elicited by exercise as well (Medina et al., 2015).

Increasingly, autophagic activity has been shown to act as a key mediator of the observed impact of CR on lifespan (Bergamini et al., 2007; Cuervo et al., 2005) with the inhibition of autophagy demonstrated to largely mitigate its longevity‐enhancing effects (Jia & Levine, 2007; Rubinsztein et al., 2011). Moreover, it has been observed that CR is capable of attenuating the impairment of autophagic activity observed in aging (Wohlgemuth, Seo, Marzetti, Lees, & Leeuwenburgh, 2010). The induction of autophagy through CR is at least partially mediated by the inhibition of TORC1 (Kenyon, 2010), which alleviates the suppressive influence TORC1 normally exerts on autophagic activity, as well as upregulating activity of AMPK (Jung et al., 2010; Meijer et al., 2015) and SIRT1 (Ma et al., 2015; Wang et al., 2011). While the mechanisms underlying SIRT1 regulation of TORC1 largely remain unclear, it is hypothesized that SIRT1 may act through interaction with tuberous sclerosis complex 2 (TSC2), a known TORC1 inhibitor (Ghosh et al., 2010; Ma et al., 2015). The relationship between AMPK and TORC1, however, is more well characterized. AMPK acts to suppress TORC1 activity in at least two ways: firstly, by activating TSC2, which prevents TORC1 from binding to a key activator, Rheb, on the lysosomal membrane (Inoki, Zhu, & Guan, 2003; Jung et al., 2010); and secondly, through direct inhibitory phosphorylation of a primary regulatory protein complex of TORC1, known as RAPTOR (Gwinn et al., 2008; Jung et al., 2010).

Some of the first data of long‐term CR on autophagic function in humans were collected from 15 lean and weight‐stable members of the Calorie Restriction Society who had practiced 30% CR for an average of 9.6 years. Upregulation of a number of autophagy modulators and gene and protein expression was noted including AMPK and SIRT family transcripts, ULK1, ATG101, APG12, GAPRAP/GATE‐6, beclin‐1, autophagin‐1, and LC3 gene expression, as well as protein expression of FOXOs, PGC1α, beclin‐1, and LC3 compared to age‐matched controls practicing a typical Western diet (Mercken et al., 2013; Yang et al., 2016).

It is also interesting to note that suppression of the TORC1 pathway has been shown to potentiate longevity beyond the maximum extension achieved with CR alone (Bjedov et al., 2010; Grandison, Piper, & Partridge, 2009). Conversely, knockdown of Atg abolishes the life‐extending effects elicited by rapamycin, suggesting a key relationship between TORC1 and autophagy with regard to aging (Bjedov et al., 2010; Rubinsztein et al., 2011), as rapamycin is a potent inhibitor of TORC1 and is known to induce autophagy under normal conditions (Kaeberlein, 2013; Xu et al., 2014). Treatment with rapamycin has been consistently shown to enhance lifespan in model species; however, the extent to which this effect is mediated by the subsequent induction of autophagy remains unclear (Kaeberlein, 2013; Pani, 2011; Rubinsztein et al., 2011; Xu et al., 2014).

## 5 EXERCISE MAY MAINTAIN THE PROTEOME

As discussed, energetic stress is a potent stimulator of autophagy; accordingly, exercise has been shown to augment acute autophagic activity in skeletal muscle (Jamart, Benoit, et al., 2012; Jamart, Francaux, et al., 2012; Tam et al., 2015; Vainshtein & Hood, 2016) as well as several other tissues including heart (He, Bassik, et al., 2012), liver (Ghareghani et al., 2017; He, Bassik, et al., 2012), pancreatic β cells (He, Bassik, et al., 2012), adipose tissue (He, Bassik, et al., 2012), peripheral blood mononuclear cells (PBMCs) (Dokladny et al., 2013), and brain (He, Sumpter, & Levine, 2012).

While some data do exist relating to other forms of autophagy (Li et al., 2016; Ulbricht et al., 2015), macroautophagy currently is the most studied and is generally the form referred to as “autophagy” within the context of exercise and training. One key function of autophagy in skeletal muscle is the provision of an emergency alternative energy source (Tam & Siu, 2014; Vainshtein, Grumati, Sandri, & Bonaldo, 2014). However, a number of other cellular challenges elicited by exercise may promote increased autophagic activity in exercised muscle as well, including widespread protein and/or mitochondrial damage, elevated mitochondrial respiration, high concentrations of reactive oxygen species (ROS), the presence of certain cytokines, and various elements of the immune response (Tam et al., 2015; Vainshtein & Hood, 2016).

During exercise, autophagy mediates the clearance of proteins and organelles damaged by heat, pH changes, or mechanical stress which likely acts to prevent accumulation of these cytosolic components and maintain myocyte function (Schwalm et al., 2015; Vainshtein et al., 2014). Moreover, alterations in calcium, NAD^+^, and ROS levels also are strong instigators of autophagic activity (Vainshtein & Hood, 2016). As such, the magnitude of the autophagic response to exercise depends in part on the extent of cellular stress and protein damage (Schwalm et al., 2015; Vainshtein & Hood, 2016). Unlike other tissues such as the liver and pancreas, upregulation of autophagy in skeletal muscle persists for days, rather than hours, following a period of energy insufficiency, indicating an elevated importance of autophagic function in skeletal muscle proteostasis (Mizushima, Yamamoto, Matsui, Yoshimori, & Ohsumi, 2004; Sandri, 2010).

In part, exercise acts to initiate autophagy in skeletal muscle through the same pathways as CR; namely, AMPK and SIRT1 are sensitive to alterations in AMP and NAD^+^, respectively. AMPK and SIRT1 both act to upregulate expression of Atgs by activating FOXO1 and FOXO3, increasing PGC1‐α activity, and inhibiting mTORC1 (Vainshtein & Hood, 2016), while AMPK also initiates autophagosome formation via ULK1 (Hardie, 2011; He, Bassik, et al., 2012; Mooren & Kruger, 2015). Though mTORC1 activity may also become reduced in response to diminished nutrient availability through ancillary pathways (Kim et al., 2016; Sarbassov, Ali, & Sabatini, 2005), the relationship between AMPK and mTORC1 is well documented (Vainshtein & Hood, 2016; Xu, Ji, & Yan, 2012). AMPK is specifically sensitive to changes in the cellular ratio of AMP to ATP and so may be strongly augmented during exercise (Hawley et al., 2014). Furthermore, exercise‐induced AMPK activation is reported to increase with increasing exercise duration (He, Bassik, et al., 2012) and intensity (Schwalm et al., 2015; Tadaishi et al., 2011). Similarly, the influence of exercise on mTORC1 activation depends in large part on the type of exercise performed, as mTORC1 integrates stimulus from growth factors, nutrient availability, and, most uniquely, mechanical loading (i.e., resistance exercise) (Goodman et al., 2011; Kim et al., 2016; Watson & Baar, 2014). While energy demands dictate a downregulation of mTORC1‐mediated anabolism during exercise that is likely affected via AMPK activation, mTORC1 activity is generally observed to be upregulated in the adaptive postexercise period, often despite a continuing elevated activity of AMPK (Kumar, Atherton, Smith, & Rennie, 2009; Rowlands et al., 2011). In addition, postexercise upregulation of the mTORC1 pathway has been shown to be potentiated by amino acid consumption following both endurance or resistance‐based exercise, highlighting the dynamic nature of mTORC1 activation (Karlsson et al., 2004; Rowlands et al., 2011). Adding to this complexity, the mTORC1 pathway seems to be independently moderated by mechanical load‐induced stress which differentiates the magnitude of mTORC1 responses to resistance versus endurance exercise (Goodman et al., 2011; Spangenburg, Le Roith, Ward, & Bodine, 2008).

mTORC1 has also been implicated in regulating autophagy activity through mediating TFEB localization which may be subsequently modulated by exercise and nutrient deprivation (Medina et al., 2015). At rest, mTORC1 phosphorylates TFEB on the lysosomal surface, confining it in the cytosol. During exercise, TFEB translocates to the nucleus as a result of the disassociation of mTORC1 from the lysosome and its dephosphorylation by Ca^++^‐dependent calcineurin where it then activates the CLEAR gene network and the transcription of Atgs and proteins.

In addition to serving as a means to meet the energetic demands of exercise, autophagy is understood to facilitate exercise in numerous ways in skeletal muscle (Dokladny et al., 2013; Grumati et al., 2011; He, Bassik, et al., 2012; Jamart, Francaux, et al., 2012; Masschelein et al., 2014; Schwalm et al., 2015). Using a mutant rodent model that inhibits exercise‐induced autophagy, He, Bassik, et al. (2012)) reported the autophagy‐deficient mice demonstrated impaired glucose uptake, GLUT4 translocation, and AMPK activation during acute exercise. Moreover, data exist suggesting autophagy possesses a role in conferring the benefits of exercise, including enhanced endurance (He, Bassik, et al., 2012; Lira et al., 2013), mitochondrial biogenesis (Grumati et al., 2011; Ju et al., 2016; Lira et al., 2013), and angiogenesis (Lira et al., 2013). Chaperone‐mediated selective autophagy has also been shown to be involved in skeletal muscle cytoskeleton maintenance and adaptation in response to resistance training (Ulbricht et al., 2015).

**Figure 2:**
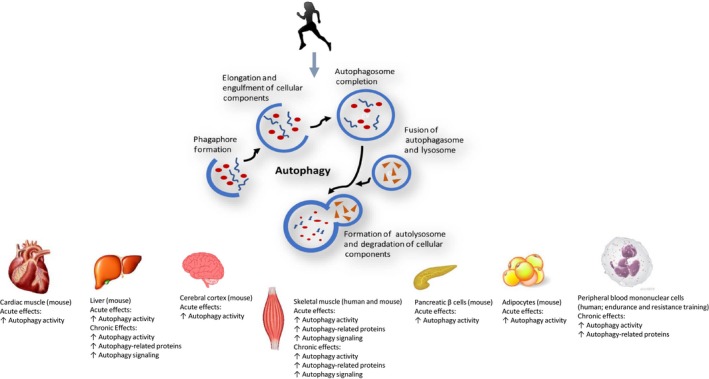
Effects of acute and chronic exercise on autophagy in multiple tissues

While exercise‐induced skeletal muscle autophagy is presently the most studied, there are data showing enhanced autophagic activity in other tissues, thus demonstrating acute exercise is capable of instigating a global autophagic response (Figure 2) (He, Bassik, et al., 2012; He, Sumpter, et al., 2012). In their study, He et al. reported acute endurance exercise increased autophagy activity in heart, liver, pancreatic β cells, and adipose tissue of wild‐type mice but not in exercise‐stimulated autophagy‐deficient mutant mice (He, Bassik, et al., 2012). Moreover, the group showed acute exercise increased autophagic flux in the anterior cerebral cortex (He, Sumpter, et al., 2012). Li and coworkers showed a number of mitochondrial autophagy (mitophagy)‐related proteins and flux were upregulated in myocardium of mice during exercise and up to 24 hr postexercise; this paralleled an increase in inflammatory markers NLRP3 and IL1β (Li et al., 2016). Additionally, expression of several Atgs was rescued in mouse hepatocytes following a high‐fat diet in response to 10 weeks of endurance exercise and was associated with reduced lipid content and lipogenic gene expression (Ghareghani et al., 2017). Further, one hour of exercise in a warm environment (30°C) increased autophagy in PBMCs (Dokladny et al., 2013). Notably, Miejas‐Pena and coworkers have shown 8 weeks of aerobic training (Mejias‐Pena et al., 2016) and 8 weeks of resistance training (Mejias‐Pena et al., 2017) augment expression in several Atgs and basal autophagic activity in PBMCs in elderly subjects.

These noted systemic autophagic effects suggest exercise could possess a role in modulating some of the age‐related pathologies that autophagy has been reported to be implicated in, which include type 2 diabetes (Gonzalez et al., 2011; Quan, Jung, & Lee, 2013), neurodegeneration (Komatsu et al., 2006; Yang et al., 2014), cardiomyopathy (Nair & Ren, 2012; Tanaka et al., 2000), cancer (Cao & Klionsky, 2007; Cecconi & Levine, 2008), and chronic inflammation (Jo, Shin, & Choi, 2012; Levine, Mizushima, & Virgin, 2011) while bolstering muscle quality and function (Fan et al., 2016; Vainshtein et al., 2014). These autophagy‐related conditions largely lie within the parameters of age‐related health benefits exercise has been documented to augment (Atherton, Phillips, & Wilkinson, 2015; Moore et al., 2016; Sanchez, Bernardi, Py, & Candau, 2014; Vainshtein et al., 2014; Woods, Wilund, Martin, & Kistler, 2012).

## 6 THE ROLE OF EXERCISE INTENSITY IN THE AUTOPHAGIC RESPONSE

Emerging evidence suggests that the autophagic response to exercise may occur in a biphasic manner in that acute cellular perturbations induce a precipitous increase in autophagic flux occurring acutely following insult and is mediated by posttranslational protein modification (Vainshtein & Hood, 2016). Moreover, autophagy appears to work in concert with another major proteolytic pathway, the ubiquitin‐proteasome system (UPS), whereby the immediate postexercise cellular degradation activity is mediated by the UPS, while autophagy activity demonstrates a more delayed response (Tam & Siu, 2014; Vainshtein & Hood, 2016); however, both systems have been shown to be activated simultaneously in some conditions (Jamart, Benoit, et al., 2012; Jamart, Francaux, et al., 2012). The exercise‐elicited autophagic response appears to be regulated in a duration and intensity‐dependent manner (Jamart, Benoit, et al., 2012; Schwalm et al., 2015; Tachtsis, Smiles, Lane, Hawley, & Camera, 2016), although an established “dose” of exercise to initiate autophagy has yet to be determined.

Aerobic exercise for 60 min or greater at 55%–70% VO_2max_ has been shown to stimulate autophagic activity in skeletal muscle (Jamart, Benoit, et al., 2012; Jamart, Francaux, et al., 2012; Moller et al., 2015; Schwalm et al., 2015). Table 1 depicts the current data of the autophagy response to acute exercise in skeletal muscle. Prolonged endurance exercise (i.e., 150 and 200 km marathon running) increased markers of autophagy and a number of related proteins in ultra‐endurance‐trained males (Jamart, Benoit, et al., 2012; Jamart, Francaux, et al., 2012). More modest bouts of exercise have also promoted an autophagic response. Cycling exercise for 60–120 min at ~50% VO_2max_ (Moller et al., 2015) and 55% and 70% VO_2peak_ (Schwalm et al., 2015) has also augmented autophagy in recreationally active and trained males, respectively.

**Table 1:** A summary of studies investigating the autophagic response to acute endurance exercise in skeletal muscle

Author	Subjects	Exercise protocol	Markers of autophagic activity	
Jamart, Benoit, et al. (2012), Jamart, Francaux, et al. (2012)	8 experienced ultra‐endurance‐trained males	200 km run (competitive race)	*3 hr post‐race:* Atg4: ↑ 40% Atg12: ↑ 57% GABARAPL1: ↑ 286% LC3B: ↑ 103% Cathespin L: ↑ 123%	BNIP3: ↑ 123% BNIP31: ↑123% beclin−1: ↔ ULK1: ↔
Jamart, Benoit, et al. (2012), Jamart, Francaux, et al. (2012)	11 experienced ultra‐endurance‐trained males	149.8 km run	*10 min postexercise* LC3B‐II: ↑ 554% cAtg12: ↑ 36% Atg7: ↔ BNIP3: ↔	beclin−1: ↔ AMPK: ↑ 247% FOXO3a: ↓ 49% mTOR: ↓ 32%
Masschelein et al. (2014)	11 healthy monozygotic twins	20 min cycling ~50% VO_2max_	*Im postexercise* LC3‐II (protein expression): ↔ LC3‐I (protein expression): ↔ LC3‐II:I (protein ratio): ↔ cATG12 (protein expression): ↔	p62 (protein expression): ↔ BNIP3 (mRNA expression): ↔ FOXO1/3a (phosphorylation): ↔ AMPK (phosphorylation): ↔
Moller et al. (2015)	8 recreationally‐ active males	60 min cycling ~50% VO_2max_	*90 min postexercise* AMPK (phosphorylation): ↑ mTOR (phosphorylation): ↔ ULK1 (phosphorylation): ↑ ULK1 (protein expression): ↔ LC3B‐II (protein expression): ↓ GABARAP (protein expression): ↓	Atg5 (protein expression): ↓ LC3B‐I (protein expression): ↔ LC3B‐II:I (protein ratio): ↓ p62 (protein expression): ↔ beclin−1 (protein expression): ↔
Tachtsis et al. (2016)	16 healthy, untrained males	60 min cycling ~70% VO_2max_	*3 hr postexercise* p53 (nuclear protein localization) ↑ Atg5 (protein expression) ↓ ULK1 (protein expression) ↔ LC3B‐I (protein expression) ↔	LC3B‐II (protein expression) ↔ LC3B‐II:I (protein ratio) ↔ p62 (protein expression) ↔
Schwalm et al. (2015)	23 trained males	2 hr cycling: 55% VO_2peak_ (fasted and fed) or 70% VO_2peak_ (fasted and fed)	*Im post, 1 hr postexercise* ULK1^Ser757^ (phosphorylation): 55% VO_2peak_ fasted: ↔ Im post; ↔ 1 hr 70% VO_2peak_ fasted: ↔ Im post; ↔ 1 hr 55% VO_2peak_ fed: ↓ Im post; ↓1 hr 70% VO_2peak_ fed: ↓Im post; ↓1 hr AMPK(phosphorylation): 55% VO_2peak_ fasted: ↔ Im post; ↔ 1 hr 70% VO_2peak_ fasted: ↑ Im post; ↔ 1 hr 55% VO_2peak_ fed: ↑ Im post; ↔ 1 hr 70% VO_2peak_ fed: ↑ Im post; ↔ 1 hr ULK1^Ser317^(phosphorylation): 55% VO_2peak_ fasted: ↑ Im post; ↔ 1 hr 70% VO_2peak_ fasted: ↑ Im post; ↑ 1 hr 55% VO_2peak_ fed: ↑ Im post; ↑ 1 hr 70% VO_2peak_ fed: ↑ Im post; ↑ 1 hr LC3B‐II (protein expression): 55% VO_2peak_ fasted: ↓ Im post; ↓ 1 hr 70% VO_2peak_ fasted: ↓ Im post; ↓ 1 hr 55% VO_2peak_ fed: ↓ Im post; ↔ 1 hr 70% VO_2peak_ fed: ↓ Im post; ↓ 1 hr LC3B‐I (protein expression): 55% VO_2peak_ fasted: ↔ Im post; ↔ 1 hr 70% VO_2peak_ fasted: ↔ Im post; ↔ 1 hr 55% VO_2peak_ fed: ↔ Im post; ↔ 1 hr 70% VO_2peak_ fed: ↔ Im post; ↔ 1 hr	*LC3B‐II:I (protein ratio):* 55% VO_2peak_ fasted: ↓ Im post; ↓ 1 hr 70% VO_2peak_ fasted: ↓ Im post; ↓ 1 hr 55% VO_2peak_ fed: ↓ Im post; ↔ 1 hr 70% VO_2peak_ fed: ↓ Im post; ↓ 1 hr p62 (mRNA expression): 55% VO_2peak_ fasted: ↔ Im post; ↔ 1 hr 70% VO_2peak_ fasted: ↔ Im post; ↓ 1 hr 55% VO_2peak_ fed: ↔ Im post; ↔ 1 hr 70% VO_2peak_ fed: ↔ Im post; ↓ 1 hr p62 (protein expression): 55% VO_2peak_ fasted: ↔ Im post; ↑ 1 hr 70% VO_2peak_ fasted: ↑ Im post; ↑ 1 hr 55% VO_2peak_ fed: ↔Im post; ↑ 1 hr 70% VO_2peak_ fed: ↑ Im post; ↑ 1 hr GABARAPL1 (mRNA exrepression): 55% VO_2peak_ fasted: ↔ Im post; ↔1 hr 70% VO_2peak_ fasted: ↑ Im post; ↑ 1 hr 55% VO_2peak_ fed: ↔Im post; ↑ 1 hr 70% VO_2peak_ fed: ↑ Im post; ↑ 1 hr Cathespin L (mRNA expression): 55% VO_2peak_ fasted: ↔ Im post; ↔ 1 hr 70% VO_2peak_ fasted: ↑ Im post; ↑ 1 hr 55% VO_2peak_ fed: ↔ Im post; ↔ 1 hr 70% VO_2peak_ fed: ↑ Im post; ↑ 1 hr

Note

hr: hour(s); Im: immediate; km: kilometers; min: minute(s); VO_2max_: maximum oxygen consumption; VO_2peak_: peak oxygen consumption.

Conversely, 20 min of cycling at ~50% VO_2max_ did not alter autophagic activity in healthy adults (Masschelein et al., 2014). Positive regulators of autophagy (AMPK and FOXO1/3a) were also unaffected, suggesting the exercise stimulus did not meet a minimum threshold of duration and/or intensity. Whereas 60 min of cycling at ~50% VO_2max_ induced autophagy (Moller et al., 2015), 60 min at 70% VO_2max_ did not produce a response (Tachtsis et al., 2016). However, this discrepancy may stem from the timing of postexercise muscle biopsies. Moller et al. performed biopsies 90 min postexercise while Tachtsis et al. performed biopsies 3 hr following exercise. Additionally, Tachtsis et al. used untrained males in their investigation, whereas Moller et al. studied recreationally trained males.

These findings help highlight the importance of exercise duration and intensity in stimulating autophagic induction and point to a threshold for activation, likely involving AMPK‐mediated determination of energy insufficiency. Importantly, the extreme elevations in autophagic activity observed with ultra‐endurance performance are likely indicative of excessive muscle damage and energetic protein catabolism, thus offering intriguing implications regarding the J‐shaped relationship observed between mortality and exercise participation (Arem et al., 2015; Kelly et al., 2014; Schnohr, O'Keefe, Marott, Lange, & Jensen, 2015). Data are needed characterizing the autophagic response to high and maximal intensity, and short duration exercise, such as high‐intensity interval training. Little data speak to the autophagic response to resistance exercise (Fry et al., 2013; Glynn et al., 2010; Smiles et al., 2015; Ulbricht et al., 2015) (Table 2) and subsequent implications on aging; however, given the role of protein turnover in response to resistance exercise, autophagy may be important.

**Table 2:** A summary of studies investigating the autophagic response to acute resistance exercise in skeletal muscle

Author	Subjects	Exercise protocol	Markers of autophagic activity	
Fry et al. (2013)	16 younger (8 females, 8 males) and 16 older (8 females, 8 males) individuals	8 sets of 10 repetitions of leg extension at 70% 1RM	*3 hr, 6 hr, and 24 hr postexercise* FOXO3a (phosphorylation): Younger: ↓ 3 hr; ↓ 6 hr; ↓24 hr Older: ↓ 3 hr; ↓ 6 hr; ↓24 hr AMPK (phosphorylation): Younger: ↔ 3 hr;↔ 6 hr; ↔ 24 hr Older: ↔ 3 hr;↔ 6 hr; ↔ 24 hr GABARAP (mRNA expression): Younger: ↓ 3 hr;↔ 6 hr; ↔ 24 hr Older: ↓ 3 hr;↔ 6 hr; ↔ 24 hr LC3B‐II (protein expression): Younger: ↔ 3 hr;↓ 6 hr; ↓24 hr Older: ↓ 3 hr; ↓ 6 hr; ↓24 hr	LC3B‐I (protein expression): Younger: ↔ 3 hr;↔ 6 hr; ↔ 24 hr Older: ↔ 3 hr;↔ 6 hr; ↔ 24 hr LC3B‐II:I (protein ratio): Younger: ↓ 3 hr; ↓ 6 hr; ↓24 hr Older: ↓ 3 hr; ↓ 6 hr; ↓24 hr Atg7 (protein expression): Younger: ↔ 3 hr;↔ 6 hr; ↔ 24 hr Older: ↔ 3 hr;↔ 6 hr; ↑ 24 hr beclin−1 (protein expression): Younger: ↔ 3 hr;↔ 6 hr; ↔ 24 hr Older: ↔ 3 hr;↔ 6 hr; ↔ 24 hr
Glynn et al. (2010)	13 young healthy males	10 sets of 10 repetitions of leg extension at 70% 1RM	*1 hr postexercise* AMPK (phosphorylation): ↑ LC3B‐II (protein expression): ↔ LC3B‐I (protein expression): ↔	
Smiles et al. (2015)	15 (8 males, 7 females) resistance‐trained individuals	6 sets of 8 repetitions at ≈80% 1RM; following 5 days of energy deficit	*1 hr, 4 hr postexercise* FOXO1 (protein expression): ↔ 1 hr; ↔ 4 hr FOXO1 (phosphorylation): ↔ 1 hr; ↔ 4 hr FOXO3a (protein expression): ↔ 1 hr; ↔ 4 hr LC3B‐I (protein expression): ↔ 1 hr; ↓ 4 hr ULK1 (phosphorylation): ↔ 1 hr; ↔ 4 hr Atg5 (protein expression): ↔ 1 hr (vs. EB); ↔ 4 hr cAtg12 (protein expression): ↔ 1 hr; ↔ 4 hr beclin−1 (protein expression): ↔ 1 hr; ↔ 4 hr p62 (protein expression): ↔ 1 hr; ↔ 4 hr	FOXO1 (mRNA expression): ↔ 1 hr; ↔ 4 hr LC3B (mRNA expression): ↔ 1 hr; ↔ 4 hr Atg12 (mRNA expression): ↔ 1 hr; ↔ 4 hr Atg4b (mRNA expression): ↔ 1 hr; ↔ 4 hr beclin−1 (mRNA expression): ↔ 1 hr; ↔ 4 hr GABARAP (mRNA expression): ↔ 1 hr; ↔ 4 hr BNIP (mRNA expression): ↔ 1 hr; ↔ 4 hr SIRT1 (mRNA expression): ↔ 1 hr; ↔ 4 hr
Ulbricht et al. (2015)	11 moderately‐trained males	3 sets of 8 ecc repetitions at 100% of max ecc force and 3 sets of 10 conc repetitions at 75% of max conc and ecc force	*15 min, 30 min, 1 hr, 4 hr, 24 hr postexercise* BAG3 (protein expression): 75% max conc and ecc: ↔ all time points 100% max ecc: ↓ 24 hr BAG3 (mRNA expression): 75% max conc and ecc: ↔ all time points 100% max ecc: ↓ 24 hr HSPB8 (protein expression): 75% max conc and ecc: ↔ all time points 100% max ecc: ↓ 24 hr HSBP8 (mRNA expression): 75% max conc and ecc: ↔ all time points 100% max ecc: ↑ 4 hr	FLNC (protein expression): 75% max conc and ecc: ↔ all time points 100% max ecc: ↓ 1 hr LC3 colocalization with FLNC and BAG3 75% max conc and ecc: ↔ 24 hr 100% max ecc: ↑ 24 hr SYNPO2 localization BAG3 75% max conc and ecc: ↔ 24 hr 100% max ecc: ↑ 24 hr

Note

1RM: one repetition maximum; conc: concentric; ecc: eccentric; hr: hour.

## 7 CHRONIC EFFECTS OF EXERCISE ON AUTOPHAGIC ACTIVITY

Currently, the long‐term effects of exercise on autophagic activity are ill‐characterized; however, they appear mediated by activation of a transcriptional program (Vainshtein & Hood, 2016). While emerging data in both rodent and human models do point to chronic exercise augmenting autophagy activity (Feng et al., 2011; Ghareghani et al., 2017; Lira et al., 2013; Mejias‐Pena et al., 2017, 2016; Wohlgemuth et al., 2011), its interaction with longevity has yet to be established. Chronic endurance exercise has long been known to promote healthy aging and mitigate age‐related disease (Arem et al., 2015; Kelly et al., 2014; Schnohr et al., 2015; Vina et al., 2016), and evidence demonstrates an inverse relationship between regular exercise and mortality (Ruiz, Moran, Arenas, & Lucia, 2011; Teramoto & Bungum, 2010; Vina et al., 2016). Longitudinal data show that physically active men and women have ~30% lower risk of death versus inactive counterparts (Schnohr et al., 2015). Moreover, highly trained individuals have been reported to have greater life expectancy. Male Finnish champion skiers lived 2.8–4.3 years longer than the general male population (Karvonen, Klemola, Virkajarvi, & Kekkonen, 1974), Tour de France cyclists from Belgium, France, and Italy had an 11% greater average longevity (Sanchis‐Gomar, Olaso‐Gonzalez, Corella, Gomez‐Cabrera, & Vina, 2011), and French cyclists had 41% lower mortality rate compared to the general male population (Marijon et al., 2013). While genetic and other lifestyle factors must undoubtedly be considered in these observations, regular exercise does appear to be associated with longevity. While an optimal “dose” of exercise for the promotion of longevity is unclear (Vina et al., 2016), so too are the mechanistic underpinnings. Considering it has been documented that CR can attenuate the age‐related impairment in autophagy (Wohlgemuth et al., 2010) and that CR and exercise share common autophagic mediators, namely AMPK, SIRT1, and recently elucidated, TFEB, it is interesting to speculate whether the observed long‐term benefits of exercise relate to mechanisms underlying the positive effects of CR on lifespan and age‐related disease, with autophagy linked to the longevity enhancements induced by both interventions (Vainshtein et al., 2014; Wohlgemuth et al., 2010).

Skeletal muscle autophagy has been studied following regular exercise. In mice, 3 months of endurance exercise has been reported to produce no changes in resting levels of LC3‐II/LC3‐I ratio within skeletal muscle (Grumati et al., 2011). Conversely, it has been reported that following 4 and 8 weeks of endurance training, markers of autophagy activity including LC3, Atg7, beclin‐1, and FOXO3 were significantly upregulated in skeletal muscle of mice (Feng et al., 2011; Lira et al., 2013). Lifelong combination of CR and exercise yielded greater skeletal muscle expression of Atg 7 and Atg 9 and LAMP‐2 mRNA abundance in mice (Wohlgemuth et al., 2010). While scant research is presently available in humans, one exploratory study in older, overweight women reported ~300% increases in Atg7, LC3, and FOXO3 mRNA expression following 6 months of moderate intensity walking and resistance training, which was accompanied by improvements in physical performance and body composition (Wohlgemuth et al., 2011).

It is also interesting to note that autophagic activity appears to be necessary for the normal adaptations of skeletal muscle (He, Bassik, et al., 2012; Lira et al., 2013; Tam et al., 2015). Recent evidence suggests that autophagy may be an important aspect of the fiber‐type shifting induced by chronic exercise, with autophagic activity preferentially upregulated in muscle fibers undergoing transition toward the oxidative phenotype (Tam et al., 2015). Additionally, mice bred to be deficient in beclin‐1 (Atg6) and saw decreased improvements in aerobic capacity with exercise training alongside decreased angiogenesis and decreased mitochondrial content (Lira et al., 2013). And mice deficient in exercise‐stimulated autophagy showed lower mitochondrial uncoupling protein 1 mRNA following 8 weeks of endurance training compared to wild‐type controls (He, Bassik, et al., 2012).

Given aging is an organismal phenomenon, it is pertinent to establish the global effects of long‐term exercise on autophagy and determine its role beyond exercised skeletal muscle. While evidences exist demonstrating acute exercise is capable of upregulating autophagic activity and/or Atg expression in a number of tissues apart from skeletal muscle including heart (He, Bassik, et al., 2012; Li et al., 2016), liver (Ghareghani et al., 2017; He, Bassik, et al., 2012), pancreatic β cells (He, Bassik, et al., 2012), adipose tissue (He, Bassik, et al., 2012), and brain (He, Sumpter, et al., 2012), limited data are currently available noting the chronic effects of exercise training in nonskeletal muscle tissues.

**Figure 3:**
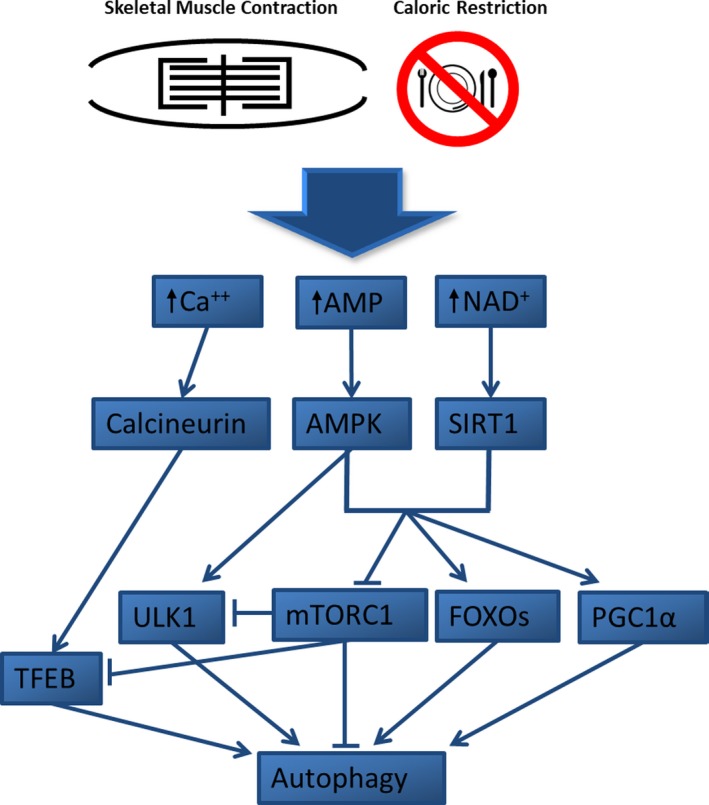
Common autophagy signaling pathways shared between skeletal muscle contraction (i.e., acute exercise) and caloric restriction. Perturbations in calcium (Ca^++^), adenosine monophosphate (AMP), and nicotinamide adenine dinucleotide (NAD^+^) activate calcineurin, 5′ adenosine monophosphate kinase (AMPK), and sirtuin‐1 (SIRT1), respectively. AMPK induces autophagosome formation through ULK1 while AMPK and SIRT1 act to upregulate expression of Atgs by increasing forkhead box transcription factors (FOXOs) and peroxisome proliferator‐activated receptor gamma coactivator 1‐alpha (PGC1α) and downregulate mammalian target of rapamycin complex 1 (mTORC1). Calcineurin activation and mTORC1 inhibition activate transcription factor EB (TFEB) which activates the CLEAR (coordinated lysosomal expression and regulation) gene network and the transcription of Atgs

Ghareghani et al. (2017) reported that 10 weeks of endurance training rescued the high‐fat diet‐induced attenuation of Atg expression in hepatocytes of mice. This occurred with a concomitant elevation of AMPK and reduced mTOR expression as well as lower lipid content and lipogenic gene expression. Miejas‐Pena and coworkers (Mejias‐Pena et al., 2016) observed an increased expression in several Atgs and basal autophagic activity in PBMCs following 8 weeks of aerobic training in elderly subjects. Additionally, He's group (He, Bassik, et al., 2012) showed favorable changes in several health parameters following 8 weeks of endurance training following a high‐fat diet in wild‐type mice versus autophagy‐deficient mutant mice, including in serum leptin, triglycerides, cholesterol, and adiponectin, glucose tolerance, basal metabolic rate, and heat production and lesser weight gain. While preliminary, these data show chronic exercise may modulate autophagic function on an organismal scale; this potentially intimates autophagy in mediating the promotion of healthy aging elicited by regular exercise (Figure 3).

## 8 CONCLUSIONS

Investigation into the mechanisms underpinning lifespan and longevity shows that the appropriate maintenance of the proteome and organelle population is key in the augmentation of lifespan and/or mitigation of many pathologies associated with the aging process (Balch, Morimoto, Dillin, & Kelly, 2008; Xu et al., 2014). Autophagy and mTORC1 represent key proteostatic pathways and are likely implicated in affecting the aging phenotype. (Rubinsztein et al., 2011; Wei et al., 2013). Moreover, autophagic function declines during aging (Cuervo & Macian, 2014; Mejias‐Pena et al., 2016; Salminen & Kaarniranta, 2012) and current investigation offers strong empirical support for the important influence exerted by autophagy over organismal lifespan (Jung et al., 2010; Madeo et al., 2015; Martinez‐Lopez et al., 2015). The similar outcomes observed with manipulation of mTORC1, in which inhibition is known to upregulate autophagic activity, provide further evidence of a potent role for autophagy in the aging process, though reductions in mTORC1 activity may also attenuate aging in an autophagy‐independent manner (Kapahi et al., 2010; Xu et al., 2014). Research exploring CR offers a particularly novel window into the impact of autophagic function and mTORC1 activity on lifespan and longevity enhancement (Madeo et al., 2015; Rubinsztein et al., 2011). In humans and rodents, acute exercise has been shown to promote autophagic activity in numerous tissues (He, Bassik, et al., 2012; Mooren & Kruger, 2015; Schwalm et al., 2015) and chronic exercise may also lead to upregulation of basal autophagy levels (Feng et al., 2011; Lira et al., 2013; Luo et al., 2013). Given that regular exercise is well evidenced to promote healthy aging and to mitigate age‐related pathologies (Bouzid et al., 2015), while sharing prominent signaling pathways with CR (Rubinsztein et al., 2011), it is interesting to speculate that the similarities in health and longevity outcomes may be traced to proteostatic maintenance as a common mediator. Currently, however, our understanding of the molecular mechanisms underlying cellular and organismal aging and the interplay between exercise and development of the aging phenotype require further study, especially in humans. Further inquiry detailing the relationship between autophagy and aging in humans, as well as potential behavioral modulators such as CR and exercise, likely represents promising means to further our understanding of human lifespan while potentially bearing application for the promotion of longevity.

## CONFLICT OF INTEREST

None declared.

## References

[acel12876-bib-0001] Alvers, A. L. , Fishwick, L. K. , Wood, M. S. , Hu, D. , Chung, H. S. , Dunn, W. A. Jr , & Aris, J. P. (2009). Autophagy and amino acid homeostasis are required for chronological longevity in *Saccharomyces cerevisiae* . Aging Cell, 8(4), 353–369. 10.1111/j.1474-9726.2009.00469.x. 19302372 PMC2802268

[acel12876-bib-0002] Arem, H. , Moore, S. C. , Patel, A. , Hartge, P. , Berrington de Gonzalez, A. , Visvanathan, K. , … Matthews, C. E. (2015). Leisure time physical activity and mortality: A detailed pooled analysis of the dose‐ response relationship. JAMA Internal Medicine, 175(6), 959–967. 10.1001/jamainternmed.2015.0533. 25844730 PMC4451435

[acel12876-bib-0003] Atherton, P. J. , Phillips, B. E. , & Wilkinson, D. J. (2015). Exercise and regulation of protein metabolism. Progress in Molecular Biology and Translational Science, 135, 75–98. 10.1016/bs.pmbts.2015.06.015. 26477911

[acel12876-bib-0004] Balch, W. E. , Morimoto, R. I. , Dillin, A. , & Kelly, J. W. (2008). Adapting proteostasis for disease intervention. Science, 319(5865), 916–919. 10.1126/science.1141448. 18276881

[acel12876-bib-0005] Barnosky, A. R. , Hoddy, K. K. , Unterman, T. G. , & Varady, K. A. (2014). Intermittent fasting vs daily calorie restriction for type 2 diabetes prevention: A review of human findings. Translational Research, 164(4), 302–311. 10.1016/j.trsl.2014.05.013. 24993615

[acel12876-bib-0006] Bergamini, E. , Cavallini, G. , Donati, A. , & Gori, Z. (2007). The role of autophagy in aging: Its essential part in the anti‐aging mechanism of caloric restriction. Annals of the New York Academy of Sciences, 1114, 69–78. 10.1196/annals.1396.020. 17934054

[acel12876-bib-0007] Bitto, A. , Ito, T. K. , Pineda, V. V. , LeTexier, N. J. , Huang, H. Z. , Sutlief, E. , … Kaeberlein, M. (2016). Transient rapamycin treatment can increase lifespan and healthspan in middle‐aged mice. eLife, 5, pii: e16351 10.7554/eLife.16351. PMC4996648 27549339

[acel12876-bib-0008] Bjedov, I. , Toivonen, J. M. , Kerr, F. , Slack, C. , Jacobson, J. , Foley, A. , & Partridge, L. (2010). Mechanisms of life span extension by rapamycin in the fruit fly *Drosophila melanogaster* . Cell Metabolism, 11(1), 35–46. 10.1016/j.cmet.2009.11.010. 20074526 PMC2824086

[acel12876-bib-0009] Booth, F. W. , Roberts, C. K. , & Laye, M. J. (2012). Lack of exercise is a major cause of chronic diseases. Comprehensive Physiology, 2(2), 1143–1211. 10.1002/cphy.c110025. 23798298 PMC4241367

[acel12876-bib-0010] Bouzid, M. A. , Filaire, E. , McCall, A. , & Fabre, C. (2015). Radical oxygen species, exercise and aging: An update. Sports Medicine (Auckland, N. Z.), 45(9), 1245–1261. 10.1007/s40279-015-0348-1. 26119427

[acel12876-bib-0011] Brandhorst, S. , Choi, I. Y. , Wei, M. , Cheng, C. W. , Sedrakyan, S. , Navarrete, G. , … Longo, V. D. (2015). A periodic diet that mimics fasting promotes multi‐system regeneration, enhanced cognitive performance, and healthspan. Cell Metabolism, 22(1), 86–99. 10.1016/j.cmet.2015.05.012. 26094889 PMC4509734

[acel12876-bib-0012] Cabreiro, F. , Au, C. , Leung, K. Y. , Vergara‐Irigaray, N. , Cocheme, H. M. , Noori, T. , … Gems, D. (2013). Metformin retards aging in *C. elegans* by altering microbial folate and methionine metabolism. Cell, 153(1), 228–239. 10.1016/j.cell.2013.02.035. 23540700 PMC3898468

[acel12876-bib-0013] Cangemi, R. , Friedmann, A. J. , Holloszy, J. O. , & Fontana, L. (2010). Long‐term effects of calorie restriction on serum sex‐hormone concentrations in men. Aging Cell, 9(2), 236–242. 10.1111/j.1474-9726.2010.00553.x. 20096034 PMC3569090

[acel12876-bib-0014] Cao, Y. , & Klionsky, D. J. (2007). Physiological functions of Atg6/Beclin 1: A unique autophagy‐related protein. Cell Research, 17(10), 839–849. 10.1038/cr.2007.78. 17893711

[acel12876-bib-0015] Carames, B. , Taniguchi, N. , Otsuki, S. , Blanco, F. J. , & Lotz, M. (2010). Autophagy is a protective mechanism in normal cartilage, and its aging‐related loss is linked with cell death and osteoarthritis. Arthritis and Rheumatism, 62(3), 791–801. 10.1002/art.27305. 20187128 PMC2838960

[acel12876-bib-0016] Cecconi, F. , & Levine, B. (2008). The role of autophagy in mammalian development: Cell makeover rather than cell death. Developmental Cell, 15(3), 344–357. 10.1016/j.devcel.2008.08.012. 18804433 PMC2688784

[acel12876-bib-0017] Chantranupong, L. , Wolfson, R. L. , Orozco, J. M. , Saxton, R. A. , Scaria, S. M. , Bar‐Peled, L. , … Sabatini, D. M. (2014). The sestrins interact with GATOR2 to negatively regulate the amino‐acid‐sensing pathway upstream of mTORC1. Cell Reports, 9(1), 1–8. 10.1016/j.celrep.2014.09.014. 25263562 PMC4223866

[acel12876-bib-0018] Colman, R. J. , Anderson, R. M. , Johnson, S. C. , Kastman, E. K. , Kosmatka, K. J. , Beasley, T. M. , … Weindruch, R. (2009). Caloric restriction delays disease onset and mortality in rhesus monkeys. Science, 325(5937), 201–204. 10.1126/science.1173635. 19590001 PMC2812811

[acel12876-bib-0019] Cuervo, A. M. , Bergamini, E. , Brunk, U. T. , Droge, W. , Ffrench, M. , & Terman, A. (2005). Autophagy and aging: The importance of maintaining "clean" cells. Autophagy, 1(3), 131–140. 10.4161/auto.1.3.2017 16874025

[acel12876-bib-0020] Cuervo, A. M. , & Macian, F. (2014). Autophagy and the immune function in aging. Current Opinion in Immunology, 29, 97–104. 10.1016/j.coi.2014.05.006. 24929664 PMC4119564

[acel12876-bib-0021] de Kreutzenberg, S. V. , Ceolotto, G. , Papparella, I. , Bortoluzzi, A. , Semplicini, A. , Dalla Man, C. , … Avogaro, A. (2010). Downregulation of the longevity‐associated protein sirtuin 1 in insulin resistance and metabolic syndrome: Potential biochemical mechanisms. Diabetes, 59(4), 1006–1015. 10.2337/db09-1187. 20068143 PMC2844808

[acel12876-bib-0022] Demontis, F. , & Perrimon, N. (2010). FOXO/4E‐BP signaling in *Drosophila* muscles regulates organism‐ wide proteostasis during aging. Cell, 143(5), 813–825. 10.1016/j.cell.2010.10.007. 21111239 PMC3066043

[acel12876-bib-0023] Dokladny, K. , Zuhl, M. N. , Mandell, M. , Bhattacharya, D. , Schneider, S. , Deretic, V. , & Moseley, P. L. (2013). Regulatory coordination between two major intracellular homeostatic systems: Heat shock response and autophagy. Journal of Biological Chemistry, 288(21), 14959–14972. 10.1074/jbc.M113.462408. 23576438 PMC3663517

[acel12876-bib-0024] Donati, A. , Recchia, G. , Cavallini, G. , & Bergamini, E. (2008). Effect of aging and anti‐aging caloric restriction on the endocrine regulation of rat liver autophagy. Journals of Gerontology. Series A, Biological Sciences and Medical Sciences, 63(6), 550–555. 10.1093/gerona/63.6.550 18559627

[acel12876-bib-0025] Donati, A. , Taddei, M. , Cavallini, G. , & Bergamini, E. (2006). Stimulation of macroautophagy can rescue older cells from 8‐OHdG mtDNA accumulation: A safe and easy way to meet goals in the SENS agenda. Rejuvenation Res, 9(3), 408–412. 10.1089/rej.2006.9.408. 16859482

[acel12876-bib-0026] Egan, D. F. , Shackelford, D. B. , Mihaylova, M. M. , Gelino, S. , Kohnz, R. A. , Mair, W. , … Shaw, R. J. (2011). Phosphorylation of ULK1 (hATG1) by AMP‐activated protein kinase connects energy sensing to mitophagy. Science, 331(6016), 456–461. 10.1126/science.1196371. 21205641 PMC3030664

[acel12876-bib-0027] Eisenberg, T. , Knauer, H. , Schauer, A. , Buttner, S. , Ruckenstuhl, C. , Carmona‐Gutierrez, D. , … Madeo, F. (2009). Induction of autophagy by spermidine promotes longevity. Nature Cell Biology, 11(11), 1305–1314. 10.1038/ncb1975. 19801973

[acel12876-bib-0028] Fabrizio, P. , Pozza, F. , Pletcher, S. D. , Gendron, C. M. , & Longo, V. D. (2001). Regulation of longevity and stress resistance by Sch9 in yeast. Science, 292(5515), 288–290. 10.1126/science.1059497. 11292860

[acel12876-bib-0029] Fan, J. , Kou, X. , Jia, S. , Yang, X. , Yang, Y. , & Chen, N. (2016). Autophagy as a potential target for sarcopenia. Journal of Cellular Physiology, 231(7), 1450–1459. 10.1002/jcp.25260. 26580995

[acel12876-bib-0030] Feng, Z. , Bai, L. , Yan, J. , Li, Y. , Shen, W. , Wang, Y. , … Liu, J. (2011). Mitochondrial dynamic remodeling in strenuous exercise‐induced muscle and mitochondrial dysfunction: Regulatory effects of hydroxytyrosol. Free Radical Biology and Medicine, 50(10), 1437–1446. 10.1016/j.freeradbiomed.2011.03.001. 21421045

[acel12876-bib-0031] Feng, Y. , He, D. , Yao, Z. , & Klionsky, D. J. (2014). The machinery of macroautophagy. Cell Research, 24(1), 24–41. 10.1038/cr.2013.168. 24366339 PMC3879710

[acel12876-bib-0032] Filfan, M. , Sandu, R. E. , Zavaleanu, A. D. , GresiTa, A. , Glavan, D. G. , Olaru, D. G. , & Popa‐Wagner, A. (2017). Autophagy in aging and disease. Romanian Journal of Morphology and Embryology, 58(1), 27–31. 28523294

[acel12876-bib-0033] Fontana, L. , & Klein, S. (2007). Aging, adiposity, and calorie restriction. JAMA, 297(9), 986–994. 10.1001/jama.297.9.986. 17341713

[acel12876-bib-0034] Fontana, L. , Meyer, T. E. , Klein, S. , & Holloszy, J. O. (2004). Long‐term calorie restriction is highly effective in reducing the risk for atherosclerosis in humans. Proceedings of the National Academy of Sciences of the United States of America, 101(17), 6659–6663. 10.1073/pnas.0308291101. 15096581 PMC404101

[acel12876-bib-0035] Fontana, L. , Partridge, L. , & Longo, V. D. (2010). Extending healthy life span–from yeast to humans. Science, 328(5976), 321–326. 10.1126/science.1172539. 20395504 PMC3607354

[acel12876-bib-0036] Fry, C. S. , Drummond, M. J. , Glynn, E. L. , Dickinson, J. M. , Gundermann, D. M. , Timmerman, K. L. , … Rasmussen, B. B. (2013). Skeletal muscle autophagy and protein breakdown following resistance exercise are similar in younger and older adults. Journals of Gerontology. Series A, Biological Sciences and Medical Sciences, 68(5), 599–607. 10.1093/gerona/gls209. PMC3623482 23089333

[acel12876-bib-0037] Ghareghani, P. , Shanaki, M. , Ahmadi, S. , Khoshdel, A. R. , Rezvan, N. , Meshkani, R. , … Gorgani‐Firuzjaee, S. (2017). Aerobic endurance training improves nonalcoholic fatty liver disease (NAFLD) features via miR‐33 dependent autophagy induction in high fat diet fed mice. Obesity Research & Clinical Practice, 10.1016/j.orcp.2017.01.004. 28163011

[acel12876-bib-0038] Ghosh, H. S. , McBurney, M. , & Robbins, P. D. (2010). SIRT1 negatively regulates the mammalian target of rapamycin. PLoS One, 5(2), e9199 10.1371/journal.pone.0009199. 20169165 PMC2821410

[acel12876-bib-0039] Glynn, E. L. , Fry, C. S. , Drummond, M. J. , Dreyer, H. C. , Dhanani, S. , Volpi, E. , & Rasmussen, B. B. (2010). Muscle protein breakdown has a minor role in the protein anabolic response to essential amino acid and carbohydrate intake following resistance exercise. American Journal of Physiology: Regulatory, Integrative and Comparative Physiology, 299(2), R533–R540. 10.1152/ajpregu.00077.2010. PMC2928613 20519362

[acel12876-bib-0040] Gonzalez, C. D. , Lee, M. S. , Marchetti, P. , Pietropaolo, M. , Towns, R. , Vaccaro, M. I. , … Wiley, J. W. (2011). The emerging role of autophagy in the pathophysiology of diabetes mellitus. Autophagy, 7(1), 2–11. 10.4161/auto.7.1.13044 20935516 PMC3359481

[acel12876-bib-0041] Goodman, C. A. , Frey, J. W. , Mabrey, D. M. , Jacobs, B. L. , Lincoln, H. C. , You, J. S. , & Hornberger, T. A. (2011). The role of skeletal muscle mTOR in the regulation of mechanical load‐induced growth. Journal of Physiology, 589(Pt 22), 5485–5501. 10.1113/jphysiol.2011.218255. 21946849 PMC3240886

[acel12876-bib-0042] Grandison, R. C. , Piper, M. D. , & Partridge, L. (2009). Amino‐acid imbalance explains extension of lifespan by dietary restriction in *Drosophila* . Nature, 462(7276), 1061–1064. 10.1038/nature08619. 19956092 PMC2798000

[acel12876-bib-0043] Grumati, P. , Coletto, L. , Schiavinato, A. , Castagnaro, S. , Bertaggia, E. , Sandri, M. , & Bonaldo, P. (2011). Physical exercise stimulates autophagy in normal skeletal muscles but is detrimental for collagen VI‐deficient muscles. Autophagy, 7(12), 1415–1423. 10.4161/auto.7.12.17877 22024752 PMC3288016

[acel12876-bib-0044] Gwinn, D. M. , Shackelford, D. B. , Egan, D. F. , Mihaylova, M. M. , Mery, A. , Vasquez, D. S. , … Shaw, R. J. (2008). AMPK phosphorylation of raptor mediates a metabolic checkpoint. Molecular Cell, 30(2), 214–226. 10.1016/j.molcel.2008.03.003. 18439900 PMC2674027

[acel12876-bib-0045] Halling, J. F. , & Pilegaard, H. (2017). Autophagy‐dependent beneficial effects of exercise. Cold Spring Harbor Perspectives in Medicine, 7(8), pii: a029777 10.1101/cshperspect.a029777. PMC5538402 28270532

[acel12876-bib-0046] Halling, J. F. , Ringholm, S. , Olesen, J. , Prats, C. , & Pilegaard, H. (2017). Exercise training protects against aging‐induced mitochondrial fragmentation in mouse skeletal muscle in a PGC‐1alpha dependent manner. Experimental Gerontology, 96, 1–6. 10.1016/j.exger.2017.05.020. 28577890

[acel12876-bib-0047] Hansen, M. , Chandra, A. , Mitic, L. L. , Onken, B. , Driscoll, M. , & Kenyon, C. (2008). A role for autophagy in the extension of lifespan by dietary restriction in *C. elegans* . PLoS Genetics, 4(2), e24 10.1371/journal.pgen.0040024. 18282106 PMC2242811

[acel12876-bib-0048] Hardie, D. G. (2011). AMPK and autophagy get connected. EMBO Journal, 30(4), 634–635. 10.1038/emboj.2011.12. 21326174 PMC3041958

[acel12876-bib-0049] Harrison, D. E. , Strong, R. , Sharp, Z. D. , Nelson, J. F. , Astle, C. M. , Flurkey, K. , … Miller, R. A. (2009). Rapamycin fed late in life extends lifespan in genetically heterogeneous mice. Nature, 460(7253), 392–395. 10.1038/nature08221. 19587680 PMC2786175

[acel12876-bib-0050] Hars, E. S. , Qi, H. , Ryazanov, A. G. , Jin, S. , Cai, L. , Hu, C. , & Liu, L. F. (2007). Autophagy regulates ageing in *C. elegans* . Autophagy, 3(2), 93–95. 17204841 10.4161/auto.3636

[acel12876-bib-0051] Hartleben, B. , Godel, M. , Meyer‐Schwesinger, C. , Liu, S. , Ulrich, T. , Kobler, S. , … Huber, T. B. (2010). Autophagy influences glomerular disease susceptibility and maintains podocyte homeostasis in aging mice. Journal of Clinical Investigation, 120(4), 1084–1096. 10.1172/JCI39492. 20200449 PMC2846040

[acel12876-bib-0052] Hawley, J. A. , Hargreaves, M. , Joyner, M. J. , & Zierath, J. R. (2014). Integrative biology of exercise. Cell, 159(4), 738–749. 10.1016/j.cell.2014.10.029. 25417152

[acel12876-bib-0053] Hawley, J. A. , & Houmard, J. A. (2004). Introduction‐preventing insulin resistance through exercise: A cellular approach. Medicine and Science in Sports and Exercise, 36(7), 1187–1190. 10.1249/01.MSS.0000132382.95142.71 15235323

[acel12876-bib-0054] He, C. , Bassik, M. C. , Moresi, V. , Sun, K. , Wei, Y. , Zou, Z. , … Levine, B. (2012). Exercise‐induced BCL2‐regulated autophagy is required for muscle glucose homeostasis. Nature, 481(7382), 511–515. 10.1038/nature10758. 22258505 PMC3518436

[acel12876-bib-0055] He, C. , Sumpter, R. Jr , & Levine, B. (2012). Exercise induces autophagy in peripheral tissues and in the brain. Autophagy, 8(10), 1548–1551. 10.4161/auto.21327. 22892563 PMC3463459

[acel12876-bib-0056] Honjoh, S. , Yamamoto, T. , Uno, M. , & Nishida, E. (2009). Signalling through RHEB‐1 mediates intermittent fasting‐induced longevity in *C. elegans* . Nature, 457(7230), 726–730. 10.1038/nature07583. 19079239

[acel12876-bib-0057] Inoki, K. , Zhu, T. , & Guan, K. L. (2003). TSC2 mediates cellular energy response to control cell growth and survival. Cell, 115(5), 577–590. 10.1016/S0092-8674(03)00929-2 14651849

[acel12876-bib-0058] Jamart, C. , Benoit, N. , Raymackers, J. M. , Kim, H. J. , Kim, C. K. , & Francaux, M. (2012). Autophagy‐related and autophagy‐regulatory genes are induced in human muscle after ultraendurance exercise. European Journal of Applied Physiology, 112(8), 3173–3177. 10.1007/s00421-011-2287-3. 22194006

[acel12876-bib-0059] Jamart, C. , Francaux, M. , Millet, G. Y. , Deldicque, L. , Frere, D. , & Feasson, L. (2012). Modulation of autophagy and ubiquitin‐proteasome pathways during ultra‐endurance running. Journal of Applied Physiology, 112(9), 1529–1537. 10.1152/japplphysiol.00952.2011. 22345427

[acel12876-bib-0060] Jia, K. , & Levine, B. (2007). Autophagy is required for dietary restriction‐mediated life span extension in *C. elegans* . Autophagy, 3(6), 597–599. 17912023 10.4161/auto.4989

[acel12876-bib-0061] Jo, E. K. , Shin, D. M. , & Choi, A. M. (2012). Autophagy: Cellular defense to excessive inflammation. Microbes and Infection, 14(2), 119–125. 10.1016/j.micinf.2011.08.014. 21924374

[acel12876-bib-0062] Johansen, T. , & Lamark, T. (2011). Selective autophagy mediated by autophagic adapter proteins. Autophagy, 7(3), 279–296. 10.4161/auto.7.3.14487 21189453 PMC3060413

[acel12876-bib-0063] Ju, J. S. , Jeon, S. I. , Park, J. Y. , Lee, J. Y. , Lee, S. C. , Cho, K. J. , & Jeong, J. M. (2016). Autophagy plays a role in skeletal muscle mitochondrial biogenesis in an endurance exercise‐trained condition. The Journal of Physiological Sciences, 66(5), 417–430. 10.1007/s12576-016-0440-9. 26943341 PMC10716990

[acel12876-bib-0064] Juhasz, G. , Erdi, B. , Sass, M. , & Neufeld, T. P. (2007). Atg7‐dependent autophagy promotes neuronal health, stress tolerance, and longevity but is dispensable for metamorphosis in *Drosophila* . Genes & Development, 21(23), 3061–3066. 10.1101/gad.1600707. 18056421 PMC2081972

[acel12876-bib-0065] Jung, C. H. , Ro, S. H. , Cao, J. , Otto, N. M. , & Kim, D. H. (2010). mTOR regulation of autophagy. FEBS Letters, 584(7), 1287–1295. 10.1016/j.febslet.2010.01.017. 20083114 PMC2846630

[acel12876-bib-0066] Kaeberlein, M. (2013). mTOR inhibition: From aging to autism and beyond. Scientifica (Cairo), 2013, 849186 10.1155/2013/849186. 24379984 PMC3860151

[acel12876-bib-0067] Kang, C. , You, Y. J. , & Avery, L. (2007). Dual roles of autophagy in the survival of *Caenorhabditis elegans* during starvation. Genes & Development, 21(17), 2161–2171. 10.1101/gad.1573107. 17785524 PMC1950855

[acel12876-bib-0068] Kapahi, P. , Chen, D. , Rogers, A. N. , Katewa, S. D. , Li, P. W. , Thomas, E. L. , & Kockel, L. (2010). With TOR, less is more: A key role for the conserved nutrient‐sensing TOR pathway in aging. Cell Metabolism, 11(6), 453–465. 10.1016/j.cmet.2010.05.001. 20519118 PMC2885591

[acel12876-bib-0069] Kapahi, P. , Zid, B. M. , Harper, T. , Koslover, D. , Sapin, V. , & Benzer, S. (2004). Regulation of lifespan in *Drosophila* by modulation of genes in the TOR signaling pathway. Current Biology, 14(10), 885–890. 10.1016/j.cub.2004.03.059. 15186745 PMC2754830

[acel12876-bib-0070] Karlsson, H. K. , Nilsson, P. A. , Nilsson, J. , Chibalin, A. V. , Zierath, J. R. , & Blomstrand, E. (2004). Branched‐ chain amino acids increase p70S6k phosphorylation in human skeletal muscle after resistance exercise. American Journal of Physiology. Endocrinology and Metabolism, 287(1), E1–E7. 10.1152/ajpendo.00430.2003. 14998784

[acel12876-bib-0071] Karvonen, M. J. , Klemola, H. , Virkajarvi, J. , & Kekkonen, A. (1974). Longevity of endurance skiers. Medicine and Science in Sports, 6(1), 49–51. 4826692

[acel12876-bib-0072] Kelly, P. , Kahlmeier, S. , Gotschi, T. , Orsini, N. , Richards, J. , Roberts, N. , … Foster, C. (2014). Systematic review and meta‐analysis of reduction in all‐cause mortality from walking and cycling and shape of dose response relationship. International Journal of Behavioral Nutrition and Physical Activity, 11, 132 10.1186/s12966-014-0132-x. 25344355 PMC4262114

[acel12876-bib-0073] Kenyon, C. J. (2010). The genetics of ageing. Nature, 464(7288), 504–512. 10.1038/nature08980. 20336132

[acel12876-bib-0074] Kim, E. , Goraksha‐Hicks, P. , Li, L. , Neufeld, T. P. , Guan, K. L. (2016). Regulation of TORC1 by Rag GTPases in nutrient response. Nature Cell Biology, 10(8), 935‐945. 10.1038/ncb1753. PMC2711503 18604198

[acel12876-bib-0075] Kim, Y. C. , & Guan, K. L. (2015). mTOR: A pharmacologic target for autophagy regulation. Journal of Clinical Investigation, 125(1), 25–32. 10.1172/JCI73939. 25654547 PMC4382265

[acel12876-bib-0076] Komatsu, M. , Waguri, S. , Chiba, T. , Murata, S. , Iwata, J. , Tanida, I. , … Tanaka, K. (2006). Loss of autophagy in the central nervous system causes neurodegeneration in mice. Nature, 441(7095), 880–884. 10.1038/nature04723. 16625205

[acel12876-bib-0077] Komatsu, M. , Waguri, S. , Ueno, T. , Iwata, J. , Murata, S. , Tanida, I. , … Chiba, T. (2005). Impairment of starvation‐induced and constitutive autophagy in Atg7‐deficient mice. Journal of Cell Biology, 169(3), 425–434. 10.1083/jcb.200412022. 15866887 PMC2171928

[acel12876-bib-0078] Kroeger, C. M. , Klempel, M. C. , Bhutani, S. , Trepanowski, J. F. , Tangney, C. C. , & Varady, K. A. (2012). Improvement in coronary heart disease risk factors during an intermittent fasting/calorie restriction regimen: Relationship to adipokine modulations. Nutrition & Metabolism, 9(1), 98 10.1186/1743-7075-9-98. 23113919 PMC3514278

[acel12876-bib-0079] Kumar, V. , Atherton, P. , Smith, K. , & Rennie, M. J. (2009). Human muscle protein synthesis and breakdown during and after exercise. Journal of Applied Physiology, 106(6), 2026–2039. 10.1152/japplphysiol.91481.2008. 19164770

[acel12876-bib-0080] Lamming, D. W. , Ye, L. , Sabatini, D. M. , & Baur, J. A. (2013). Rapalogs and mTOR inhibitors as anti‐aging therapeutics. Journal of Clinical Investigation, 123(3), 980–989. 10.1172/JCI64099. 23454761 PMC3582126

[acel12876-bib-0081] Laplante, M. , & Sabatini, D. M. (2012). mTOR signaling in growth control and disease. Cell, 149(2), 274–293. 10.1016/j.cell.2012.03.017. 22500797 PMC3331679

[acel12876-bib-0082] Lee, J. H. , Budanov, A. V. , Park, E. J. , Birse, R. , Kim, T. E. , Perkins, G. A. , … Karin, M. (2010). Sestrin as a feedback inhibitor of TOR that prevents age‐related pathologies. Science, 327(5970), 1223–1228. 10.1126/science.1182228. 20203043 PMC2866632

[acel12876-bib-0083] Levine, B. , Mizushima, N. , & Virgin, H. W. (2011). Autophagy in immunity and inflammation. Nature, 469(7330), 323–335. 10.1038/nature09782. 21248839 PMC3131688

[acel12876-bib-0084] Li, H. , Miao, W. , Ma, J. , Xv, Z. , Bo, H. , Li, J. , … Ji, L. L. (2016). Acute exercise‐induced mitochondrial stress triggers an inflammatory response in the myocardium via NLRP3 inflammasome activation with mitophagy. Oxidative Medicine and Cellular Longevity, 2016, 1987149 10.1155/2016/1987149. 26770647 PMC4684864

[acel12876-bib-0085] Li, F. , & Vierstra, R. D. (2012). Autophagy: A multifaceted intracellular system for bulk and selective recycling. Trends in Plant Science, 17(9), 526–537. 10.1016/j.tplants.2012.05.006. 22694835

[acel12876-bib-0086] Liang, C. , & Jung, J. U. (2010). Autophagy genes as tumor suppressors. Current Opinion in Cell Biology, 22(2), 226–233. 10.1016/j.ceb.2009.11.003. 19945837 PMC2854193

[acel12876-bib-0087] Liang, C. C. , Wang, C. , Peng, X. , Gan, B. , & Guan, J. L. (2010). Neural‐specific deletion of FIP200 leads to cerebellar degeneration caused by increased neuronal death and axon degeneration. Journal of Biological Chemistry, 285(5), 3499–3509. 10.1074/jbc.M109.072389. 19940130 PMC2823459

[acel12876-bib-0088] Lipinski, M. M. , Zheng, B. , Lu, T. , Yan, Z. , Py, B. F. , Ng, A. , … Yuan, J. (2010). Genome‐wide analysis reveals mechanisms modulating autophagy in normal brain aging and in Alzheimer's disease. Proceedings of the National Academy of Sciences of the United States of America, 107(32), 14164–14169. 10.1073/pnas.1009485107. 20660724 PMC2922576

[acel12876-bib-0089] Lira, V. A. , Okutsu, M. , Zhang, M. , Greene, N. P. , Laker, R. C. , Breen, D. S. , … Yan, Z. (2013). Autophagy is required for exercise training‐induced skeletal muscle adaptation and improvement of physical performance. The FASEB Journal, 27(10), 4184–4193. 10.1096/fj.13-228486. 23825228 PMC4046188

[acel12876-bib-0090] Lopez‐Otin, C. , Blasco, M. A. , Partridge, L. , Serrano, M. , & Kroemer, G. (2013). The hallmarks of aging. Cell, 153(6), 1194‐1217. 10.1016/j.cell.2013.05.039 23746838 PMC3836174

[acel12876-bib-0091] Luo, L. , Lu, A. M. , Wang, Y. , Hong, A. , Chen, Y. , Hu, J. , … Qin, Z. H. (2013). Chronic resistance training activates autophagy and reduces apoptosis of muscle cells by modulating IGF‐1 and its receptors, Akt/mTOR and Akt/FOXO3a signaling in aged rats. Experimental Gerontology, 48(4), 427–436. 10.1016/j.exger.2013.02.009. 23419688

[acel12876-bib-0092] Ma, L. , Dong, W. , Wang, R. , Li, Y. , Xu, B. , Zhang, J. , … Wang, Y. (2015). Effect of caloric restriction on the SIRT1/mTOR signaling pathways in senile mice. Brain Research Bulletin, 116, 67–72. 10.1016/j.brainresbull.2015.06.004. 26135885

[acel12876-bib-0093] Madeo, F. , Tavernarakis, N. , & Kroemer, G. (2010). Can autophagy promote longevity? Nature Cell Biology, 12(9), 842–846. 10.1038/ncb0910-842. 20811357

[acel12876-bib-0094] Madeo, F. , Zimmermann, A. , Maiuri, M. C. , & Kroemer, G. (2015). Essential role for autophagy in life span extension. Journal of Clinical Investigation, 125(1), 85–93. 10.1172/JCI73946. 25654554 PMC4382258

[acel12876-bib-0095] Marijon, E. , Tafflet, M. , Antero‐Jacquemin, J. , El Helou, N. , Berthelot, G. , Celermajer, D. S. , … Jouven, X. (2013). Mortality of French participants in the Tour de France (1947–2012). European Heart Journal, 34(40), 3145–3150. 10.1093/eurheartj/eht347. 24001718

[acel12876-bib-0096] Martin, B. , Mattson, M. P. , & Maudsley, S. (2006). Caloric restriction and intermittent fasting: Two potential diets for successful brain aging. Ageing Research Reviews, 5(3), 332–353. 10.1016/j.arr.2006.04.002. 16899414 PMC2622429

[acel12876-bib-0097] Martina, J. A. , Chen, Y. , Gucek, M. , & Puertollano, R. (2012). MTORC1 functions as a transcriptional regulator of autophagy by preventing nuclear transport of TFEB. Autophagy, 8(6), 903–914. 10.4161/auto.19653. 22576015 PMC3427256

[acel12876-bib-0098] Martinez‐Lopez, N. , Athonvarangkul, D. , & Singh, R. (2015). Autophagy and aging. Advances in Experimental Medicine and Biology, 847, 73–87. 10.1007/978-1-4939-2404-2_3. 25916586 PMC4644734

[acel12876-bib-0099] Masiero, E. , Agatea, L. , Mammucari, C. , Blaauw, B. , Loro, E. , Komatsu, M. , … Sandri, M. (2009). Autophagy is required to maintain muscle mass. Cell Metabolism, 10(6), 507–515. 10.1016/j.cmet.2009.10.008. 19945408

[acel12876-bib-0100] Masschelein, E. , Van Thienen, R. , D'Hulst, G. , Hespel, P. , Thomis, M. , & Deldicque, L. (2014). Acute environmental hypoxia induces LC3 lipidation in a genotype‐dependent manner. The FASEB Journal, 28(2), 1022–1034. 10.1096/fj.13-239863. 24200883

[acel12876-bib-0101] Medina, D. L. , Di Paola, S. , Peluso, I. , Armani, A. , De Stefani, D. , Venditti, R. , … Ballabio, A. (2015). Lysosomal calcium signalling regulates autophagy through calcineurin and TFEB. Nature Cell Biology, 17(3), 288–299. 10.1038/ncb3114. 25720963 PMC4801004

[acel12876-bib-0102] Meijer, A. J. , Lorin, S. , Blommaart, E. F. , & Codogno, P. (2015). Regulation of autophagy by amino acids and MTOR‐dependent signal transduction. Amino Acids, 47(10), 2037–2063. 10.1007/s00726-014-1765-4. 24880909 PMC4580722

[acel12876-bib-0103] Mejias‐Pena, Y. , Estebanez, B. , Rodriguez‐Miguelez, P. , Fernandez‐Gonzalo, R. , Almar, M. , de Paz, J. A. , & Cuevas, M. J. (2017). Impact of resistance training on the autophagy‐inflammation‐apoptosis crosstalk in elderly subjects. Aging (Albany NY), 10.18632/aging.101167. PMC5361672 28160545

[acel12876-bib-0104] Mejias‐Pena, Y. , Rodriguez‐Miguelez, P. , Fernandez‐Gonzalo, R. , Martinez‐Florez, S. , Almar, M. , de Paz, J. A. , … Gonzalez‐Gallego, J. (2016). Effects of aerobic training on markers of autophagy in the elderly. Age (Dordr), 38(2), 33 10.1007/s11357-016-9897-y. 26940016 PMC5005904

[acel12876-bib-0105] Melendez, A. , Talloczy, Z. , Seaman, M. , Eskelinen, E. L. , Hall, D. H. , & Levine, B. (2003). Autophagy genes are essential for dauer development and life‐span extension in *C. elegans* . Science, 301(5638), 1387–1391. 10.1126/science.1087782. 12958363

[acel12876-bib-0106] Mercken, E. M. , Crosby, S. D. , Lamming, D. W. , JeBailey, L. , Krzysik‐Walker, S. , Villareal, D. T. , … Fontana, L. (2013). Calorie restriction in humans inhibits the PI3K/AKT pathway and induces a younger transcription profile. Aging Cell, 12(4), 645–651. 10.1111/acel.12088. 23601134 PMC3714316

[acel12876-bib-0107] Meyer, T. E. , Kovacs, S. J. , Ehsani, A. A. , Klein, S. , Holloszy, J. O. , & Fontana, L. (2006). Long‐term caloric restriction ameliorates the decline in diastolic function in humans. Journal of the American College of Cardiology, 47(2), 398–402. 10.1016/j.jacc.2005.08.069. 16412867

[acel12876-bib-0108] Mirzaei, H. , Suarez, J. A. , & Longo, V. D. (2014). Protein and amino acid restriction, aging and disease: From yeast to humans. Trends in Endocrinology and Metabolism, 25(11), 558–566. 10.1016/j.tem.2014.07.002. 25153840 PMC4254277

[acel12876-bib-0109] Mizushima, N. , Yamamoto, A. , Matsui, M. , Yoshimori, T. , & Ohsumi, Y. (2004). In vivo analysis of autophagy in response to nutrient starvation using transgenic mice expressing a fluorescent autophagosome marker. Molecular Biology of the Cell, 15(3), 1101–1111. 10.1091/mbc.E03-09-0704. 14699058 PMC363084

[acel12876-bib-0110] Moller, A. B. , Vendelbo, M. H. , Christensen, B. , Clasen, B. F. , Bak, A. M. , Jorgensen, J. O. , … Jessen, N. (2015). Physical exercise increases autophagic signaling through ULK1 in human skeletal muscle. Journal of Applied Physiology (1985), 118(8), 971–979. 10.1152/japplphysiol.01116.2014. 25678702

[acel12876-bib-0111] Moore, S. C. , Lee, I. M. , Weiderpass, E. , Campbell, P. T. , Sampson, J. N. , Kitahara, C. M. , … Patel, A. V. (2016). Association of leisure‐time physical activity with risk of 26 types of cancer in 1.44 million adults. JAMA Internal Medicine, 176(6), 816–825. 10.1001/jamainternmed.2016.1548. 27183032 PMC5812009

[acel12876-bib-0112] Mooren, F. C. , & Kruger, K. (2015). Exercise, autophagy, and apoptosis. Progress in Molecular Biology and Translational Science, 135, 407–422. 10.1016/bs.pmbts.2015.07.023. 26477924

[acel12876-bib-0113] Morselli, E. , Maiuri, M. C. , Markaki, M. , Megalou, E. , Pasparaki, A. , Palikaras, K. , … Kroemer, G. (2010). Caloric restriction and resveratrol promote longevity through the Sirtuin‐1‐dependent induction of autophagy. Cell Death & Disease, 1, e10 10.1038/cddis.2009.8. 21364612 PMC3032517

[acel12876-bib-0114] Most, J. , Tosti, V. , Redman, L. M. , & Fontana, L. (2016). Calorie restriction in humans: An update. Ageing Research Reviews, 10.1016/j.arr.2016.08.005. PMC5315691 27544442

[acel12876-bib-0115] Mouchiroud, L. , Molin, L. , Dalliere, N. , & Solari, F. (2010). Life span extension by resveratrol, rapamycin, and metformin: The promise of dietary restriction mimetics for an healthy aging. BioFactors, 36(5), 377–382. 10.1002/biof.127. 20848587

[acel12876-bib-0116] Nair, S. , & Ren, J. (2012). Autophagy and cardiovascular aging: Lesson learned from rapamycin. Cell Cycle, 11(11), 2092–2099. 10.4161/cc.20317. 22580468 PMC3368861

[acel12876-bib-0117] Ng, F. , & Tang, B. L. (2013). Sirtuins' modulation of autophagy. Journal of Cellular Physiology, 228(12), 2262–2270. 10.1002/jcp.24399. 23696314

[acel12876-bib-0118] Palmieri, M. , Impey, S. , Kang, H. , di Ronza, A. , Pelz, C. , Sardiello, M. , & Ballabio, A. (2011). Characterization of the CLEAR network reveals an integrated control of cellular clearance pathways. Human Molecular Genetics, 20(19), 3852–3866. 10.1093/hmg/ddr306. 21752829

[acel12876-bib-0119] Pan, K. Z. , Palter, J. E. , Rogers, A. N. , Olsen, A. , Chen, D. , Lithgow, G. J. , & Kapahi, P. (2007). Inhibition of mRNA translation extends lifespan in *Caenorhabditis elegans* . Aging Cell, 6(1), 111–119. 10.1111/j.1474-9726.2006.00266.x. 17266680 PMC2745345

[acel12876-bib-0120] Pani, G. (2011). From growing to secreting: New roles for mTOR in aging cells. Cell Cycle, 10(15), 2450–2453. 10.4161/cc.10.15.16886. 21720215

[acel12876-bib-0121] Phadwal, K. , Alegre‐Abarrategui, J. , Watson, A. S. , Pike, L. , Anbalagan, S. , Hammond, E. M. , … Simon, A. K. (2012). A novel method for autophagy detection in primary cells: Impaired levels of macroautophagy in immunosenescent T cells. Autophagy, 8(4), 677–689. 10.4161/auto.18935. 22302009 PMC3405842

[acel12876-bib-0122] Pyo, J. O. , Yoo, S. M. , Ahn, H. H. , Nah, J. , Hong, S. H. , Kam, T. I. , … Jung, Y. K. (2013). Overexpression of Atg5 in mice activates autophagy and extends lifespan. Nature Communications, 4, 2300 10.1038/ncomms3300. PMC3753544 23939249

[acel12876-bib-0123] Quan, W. , Jung, H. S. , & Lee, M. S. (2013). Role of autophagy in the progression from obesity to diabetes and in the control of energy balance. Archives of Pharmacal Research, 36(2), 223–229. 10.1007/s12272-013-0024-7. 23371805

[acel12876-bib-0124] Rajawat, Y. S. , & Bossis, I. (2008). Autophagy in aging and in neurodegenerative disorders. Hormones (Athens), 7(1), 46–61. 10.14310/horm.2002.1111037 18359744

[acel12876-bib-0125] Rajawat, Y. S. , Hilioti, Z. , & Bossis, I. (2009). Aging: Central role for autophagy and the lysosomal degradative system. Ageing Research Reviews, 8(3), 199–213. 10.1016/j.arr.2009.05.001. 19427410

[acel12876-bib-0126] Rowlands, D. S. , Thomson, J. S. , Timmons, B. W. , Raymond, F. , Fuerholz, A. , Mansourian, R. , … Tarnopolsky, M. A. (2011). Transcriptome and translational signaling following endurance exercise in trained skeletal muscle: Impact of dietary protein. Physiological Genomics, 43(17), 1004–1020. 10.1152/physiolgenomics.00073.2011. 21730029

[acel12876-bib-0127] Rubinsztein, D. C. , Marino, G. , & Kroemer, G. (2011). Autophagy and aging. Cell, 146(5), 682–695. 10.1016/j.cell.2011.07.030. 21884931

[acel12876-bib-0128] Ruiz, J. R. , Moran, M. , Arenas, J. , & Lucia, A. (2011). Strenuous endurance exercise improves life expectancy: It's in our genes. British Journal of Sports Medicine, 45(3), 159–161. 10.1136/bjsm.2010.075085. 20876590

[acel12876-bib-0129] Salminen, A. , & Kaarniranta, K. (2009). Regulation of the aging process by autophagy. Trends in Molecular Medicine, 15(5), 217–224. 10.1016/j.molmed.2009.03.004. 19380253

[acel12876-bib-0130] Salminen, A. , & Kaarniranta, K. (2012). AMP‐activated protein kinase (AMPK) controls the aging process via an integrated signaling network. Ageing Research Reviews, 11(2), 230–241. 10.1016/j.arr.2011.12.005. 22186033

[acel12876-bib-0131] Sanchez, A. M. , Bernardi, H. , Py, G. , & Candau, R. B. (2014). Autophagy is essential to support skeletal muscle plasticity in response to endurance exercise. American Journal of Physiology: Regulatory, Integrative and Comparative Physiology, 307(8), R956–R969. 10.1152/ajpregu.00187.2014. 25121614

[acel12876-bib-0132] Sanchis‐Gomar, F. , Olaso‐Gonzalez, G. , Corella, D. , Gomez‐Cabrera, M. C. , & Vina, J. (2011). Increased average longevity among the "Tour de France" cyclists. International Journal of Sports Medicine, 32(8), 644–647. 10.1055/s-0031-1271711. 21618162

[acel12876-bib-0133] Sandri, M. (2010). Autophagy in skeletal muscle. FEBS Letters, 584(7), 1411–1416. 10.1016/j.febslet.2010.01.056. 20132819

[acel12876-bib-0134] Sarbassov, D. D. , Ali, S. M. , Kim, D. H. , Guertin, D. A. , Latek, R. R. , Erdjument‐Bromage, H. , … Sabatini, D. M. (2004). Rictor, a novel binding partner of mTOR, defines a rapamycin‐insensitive and raptor‐ independent pathway that regulates the cytoskeleton. Current Biology, 14(14), 1296–1302. 10.1016/j.cub.2004.06.054. 15268862

[acel12876-bib-0135] Sarbassov, D. D. , Ali, S. M. , & Sabatini, D. M. (2005). Growing roles for the mTOR pathway. Current Opinion in Cell Biology, 17(6), 596–603. 10.1016/j.ceb.2005.09.009. 16226444

[acel12876-bib-0136] Sarbassov, D. D. , Ali, S. M. , Sengupta, S. , Sheen, J. H. , Hsu, P. P. , Bagley, A. F. , … Sabatini, D. M. (2006). Prolonged rapamycin treatment inhibits mTORC2 assembly and Akt/PKB. Molecular Cell, 22(2), 159–168. 10.1016/j.molcel.2006.03.029. 16603397

[acel12876-bib-0137] Sarbassov, D. D. , Guertin, D. A. , Ali, S. M. , & Sabatini, D. M. (2005). Phosphorylation and regulation of Akt/PKB by the rictor‐mTOR complex. Science, 307(5712), 1098–1101. 10.1126/science.1106148. 15718470

[acel12876-bib-0138] Sardiello, M. , Palmieri, M. , di Ronza, A. , Medina, D. L. , Valenza, M. , Gennarino, V. A. , … Ballabio, A. (2009). A gene network regulating lysosomal biogenesis and function. Science, 325(5939), 473–477. 10.1126/science.1174447. 19556463

[acel12876-bib-0139] Schnohr, P. , O'Keefe, J. H. , Marott, J. L. , Lange, P. , & Jensen, G. B. (2015). Dose of jogging and long‐term mortality: The Copenhagen City Heart Study. Journal of the American College of Cardiology, 65(5), 411–419. 10.1016/j.jacc.2014.11.023. 25660917

[acel12876-bib-0140] Schwalm, C. , Jamart, C. , Benoit, N. , Naslain, D. , Premont, C. , Prevet, J. , … Francaux, M. (2015). Activation of autophagy in human skeletal muscle is dependent on exercise intensity and AMPK activation. The FASEB Journal, 29(8), 3515–3526. 10.1096/fj.14-267187. 25957282

[acel12876-bib-0141] Selman, C. , Tullet, J. M. , Wieser, D. , Irvine, E. , Lingard, S. J. , Choudhury, A. I. , … Withers, D. J. (2009). Ribosomal protein S6 kinase 1 signaling regulates mammalian life span. Science, 326(5949), 140–144. 10.1126/science.1177221. 19797661 PMC4954603

[acel12876-bib-0142] Settembre, C. , Di Malta, C. , Polito, V. A. , Garcia Arencibia, M. , Vetrini, F. , Erdin, S. , … Ballabio, A. (2011). TFEB links autophagy to lysosomal biogenesis. Science, 332(6036), 1429–1433. 10.1126/science.1204592. 21617040 PMC3638014

[acel12876-bib-0143] Simonsen, A. , Cumming, R. C. , Brech, A. , Isakson, P. , Schubert, D. R. , & Finley, K. D. (2008). Promoting basal levels of autophagy in the nervous system enhances longevity and oxidant resistance in adult *Drosophila* . Autophagy, 4(2), 176–184. 10.4161/auto.5269 18059160

[acel12876-bib-0144] Smiles, W. J. , Areta, J. L. , Coffey, V. G. , Phillips, S. M. , Moore, D. R. , Stellingwerff, T. , … Camera, D. M. (2015). Modulation of autophagy signaling with resistance exercise and protein ingestion following short‐term energy deficit. American Journal of Physiology: Regulatory, Integrative and Comparative Physiology, 309(5), R603–R612. 10.1152/ajpregu.00413.2014. 26136534

[acel12876-bib-0145] Spangenburg, E. E. , Le Roith, D. , Ward, C. W. , & Bodine, S. C. (2008). A functional insulin‐like growth factor receptor is not necessary for load‐induced skeletal muscle hypertrophy. Journal of Physiology, 586(1), 283–291. 10.1113/jphysiol.2007.141507. 17974583 PMC2375552

[acel12876-bib-0146] Stein, P. K. , Soare, A. , Meyer, T. E. , Cangemi, R. , Holloszy, J. O. , & Fontana, L. (2012). Caloric restriction may reverse age‐related autonomic decline in humans. Aging Cell, 11(4), 644–650. 10.1111/j.1474-9726.2012.00825.x. 22510429 PMC3598611

[acel12876-bib-0147] Tachtsis, B. , Smiles, W. J. , Lane, S. C. , Hawley, J. A. , & Camera, D. M. (2016). Acute endurance exercise induces nuclear p53 abundance in human skeletal muscle. Frontiers in Physiology, 7, 144 10.3389/fphys.2016.00144. 27199762 PMC4845512

[acel12876-bib-0148] Tadaishi, M. , Miura, S. , Kai, Y. , Kawasaki, E. , Koshinaka, K. , Kawanaka, K. , … Ezaki, O. (2011). Effect of exercise intensity and AICAR on isoform‐specific expressions of murine skeletal muscle PGC‐ 1alpha mRNA: A role of beta(2)‐adrenergic receptor activation. American Journal of Physiology. Endocrinology and Metabolism, 300(2), E341–E349. 10.1152/ajpendo.00400.2010. 21098736

[acel12876-bib-0149] Tam, B. T. , Pei, X. M. , Yu, A. P. , Sin, T. K. , Leung, K. K. , Au, K. K. , … Siu, P. M. (2015). Autophagic adaptation is associated with exercise‐induced fibre‐type shifting in skeletal muscle. Acta Psychologica, 214(2), 221–236. 10.1111/apha.12503. 25847142

[acel12876-bib-0150] Tam, B. T. , & Siu, P. M. (2014). Autophagic cellular responses to physical exercise in skeletal muscle. Sports Medicine (Auckland, N. Z.), 44(5), 625–640. 10.1007/s40279-013-0140-z. 24549475

[acel12876-bib-0151] Tanaka, Y. , Guhde, G. , Suter, A. , Eskelinen, E. L. , Hartmann, D. , Lullmann‐Rauch, R. , … Saftig, P. (2000). Accumulation of autophagic vacuoles and cardiomyopathy in LAMP‐2‐deficient mice. Nature, 406(6798), 902–906. 10.1038/35022595. 10972293

[acel12876-bib-0152] Teramoto, M. , & Bungum, T. J. (2010). Mortality and longevity of elite athletes. Journal of Science and Medicine in Sport, 13(4), 410–416. 10.1016/j.jsams.2009.04.010. 19574095

[acel12876-bib-0153] Toth, M. L. , Sigmond, T. , Borsos, E. , Barna, J. , Erdelyi, P. , Takacs‐Vellai, K. , … Vellai, T. (2008). Longevity pathways converge on autophagy genes to regulate life span in *Caenorhabditis elegans* . Autophagy, 4(3), 330–338. 18219227 10.4161/auto.5618

[acel12876-bib-0154] Ulbricht, A. , Gehlert, S. , Leciejewski, B. , Schiffer, T. , Bloch, W. , & Hohfeld, J. (2015). Induction and adaptation of chaperone‐assisted selective autophagy CASA in response to resistance exercise in human skeletal muscle. Autophagy, 11(3), 538–546. 10.1080/15548627.2015.1017186. 25714469 PMC4502687

[acel12876-bib-0155] Vainshtein, A. , Grumati, P. , Sandri, M. , & Bonaldo, P. (2014). Skeletal muscle, autophagy, and physical activity: The menage a trois of metabolic regulation in health and disease. Journal of Molecular Medicine (Berlin), 92(2), 127–137. 10.1007/s00109-013-1096-z. 24271008

[acel12876-bib-0156] Vainshtein, A. , & Hood, D. A. (2016). The regulation of autophagy during exercise in skeletal muscle. Journal of Applied Physiology (1985), 120(6), 664–673. 10.1152/japplphysiol.00550.2015. PMC4796178 26679612

[acel12876-bib-0157] Vellai, T. , Takacs‐Vellai, K. , Zhang, Y. , Kovacs, A. L. , Orosz, L. , & Muller, F. (2003). Genetics: Influence of TOR kinase on lifespan in *C. elegans* . Nature, 426(6967), 620 10.1038/426620a. 14668850

[acel12876-bib-0158] Vina, J. , Rodriguez‐Manas, L. , Salvador‐Pascual, A. , Tarazona‐Santabalbina, F. J. , & Gomez‐Cabrera, M. C. (2016). Exercise: The lifelong supplement for healthy ageing and slowing down the onset of frailty. Journal of Physiology, 594(8), 1989–1999. 10.1113/JP270536. 26872560 PMC4933124

[acel12876-bib-0159] Wang, Y. , Liang, Y. , & Vanhoutte, P. M. (2011). SIRT1 and AMPK in regulating mammalian senescence: A critical review and a working model. FEBS Letters, 585(7), 986–994. 10.1016/j.febslet.2010.11.047. 21130086

[acel12876-bib-0160] Wang, X. , Zuo, X. , Kucejova, B. , & Chen, X. J. (2008). Reduced cytosolic protein synthesis suppresses mitochondrial degeneration. Nature Cell Biology, 10(9), 1090–1097. 10.1038/ncb1769 19160490 PMC2762220

[acel12876-bib-0161] Watson, K. , & Baar, K. (2014). mTOR and the health benefits of exercise. Seminars in Cell & Developmental Biology, 36, 130–139. 10.1016/j.semcdb.2014.08.013. 25218794

[acel12876-bib-0162] Wei, M. , Brandhorst, S. , Shelehchi, M. , Mirzaei, H. , Cheng, C. W. , Budniak, J. , … Longo, V. D. (2017). Fasting‐mimicking diet and markers/risk factors for aging, diabetes, cancer, and cardiovascular disease. Science Translational Medicine, 9(377), pii: eaai8700. doi:10.1126/scitranslmed.aai8700. PMC6816332 28202779

[acel12876-bib-0163] Wei, Y. , Zhang, Y. J. , & Cai, Y. (2013). Growth or longevity: The TOR's decision on lifespan regulation. Biogerontology, 14(4), 353–363. 10.1007/s10522-013-9435-6. 23740528

[acel12876-bib-0164] Weindruch, R. , Walford, R. L. , Fligiel, S. , & Guthrie, D. (1986). The retardation of aging in mice by dietary restriction: Longevity, cancer, immunity and lifetime energy intake. Journal of Nutrition, 116(4), 641–654. 10.1093/jn/116.4.641 3958810

[acel12876-bib-0165] Wohlgemuth, S. E. , Lees, H. A. , Marzetti, E. , Manini, T. M. , Aranda, J. M. , Daniels, M. J. , … Anton, S. D. (2011). An exploratory analysis of the effects of a weight loss plus exercise program on cellular quality control mechanisms in older overweight women. Rejuvenation Research, 14(3), 315–324. 10.1089/rej.2010.1132. 21631380 PMC3136739

[acel12876-bib-0166] Wohlgemuth, S. E. , Seo, A. Y. , Marzetti, E. , Lees, H. A. , & Leeuwenburgh, C. (2010). Skeletal muscle autophagy and apoptosis during aging: Effects of calorie restriction and life‐long exercise. Experimental Gerontology, 45(2), 138–148. 10.1016/j.exger.2009.11.002. 19903516 PMC2829942

[acel12876-bib-0167] Woods, J. A. , Wilund, K. R. , Martin, S. A. , & Kistler, B. M. (2012). Exercise, inflammation and aging. Aging and Disease, 3(1), 130–140. 22500274 PMC3320801

[acel12876-bib-0168] Wu, J. J. , Liu, J. , Chen, E. B. , Wang, J. J. , Cao, L. , Narayan, N. , … Finkel, T. (2013). Increased mammalian lifespan and a segmental and tissue‐specific slowing of aging after genetic reduction of mTOR expression. Cell Reports, 4(5), 913–920. 10.1016/j.celrep.2013.07.030. 23994476 PMC3784301

[acel12876-bib-0169] Wu, J. J. , Quijano, C. , Chen, E. , Liu, H. , Cao, L. , Fergusson, M. M. , … Finkel, T. (2009). Mitochondrial dysfunction and oxidative stress mediate the physiological impairment induced by the disruption of autophagy. Aging (Albany NY), 1(4), 425–437. 10.18632/aging.100038. 20157526 PMC2806022

[acel12876-bib-0170] Xu, S. , Cai, Y. , & Wei, Y. (2014). mTOR signaling from cellular senescence to organismal aging. Aging and Disease, 5(4), 263–273. 10.14336/AD.2014.0500263. 25110610 PMC4113516

[acel12876-bib-0171] Xu, J. , Ji, J. , & Yan, X. H. (2012). Cross‐talk between AMPK and mTOR in regulating energy balance. Critical Reviews in Food Science and Nutrition, 52(5), 373–381. 10.1080/10408398.2010.500245. 22369257

[acel12876-bib-0172] Yang, F. , Chu, X. , Yin, M. , Liu, X. , Yuan, H. , Niu, Y. , & Fu, L. (2014). mTOR and autophagy in normal brain aging and caloric restriction ameliorating age‐related cognition deficits. Behavioral Brain Research, 264, 82–90. 10.1016/j.bbr.2014.02.005. 24525424

[acel12876-bib-0173] Yang, L. , Licastro, D. , Cava, E. , Veronese, N. , Spelta, F. , Rizza, W. , … Fontana, L. (2016). Long‐term calorie restriction enhances cellular quality‐control processes in human skeletal muscle. Cell Reports, 14(3), 422–428. 10.1016/j.celrep.2015.12.042. 26774472

[acel12876-bib-0174] Zid, B. M. , Rogers, A. N. , Katewa, S. D. , Vargas, M. A. , Kolipinski, M. C. , Lu, T. A. , … Kapahi, P. (2009). 4E‐BP extends lifespan upon dietary restriction by enhancing mitochondrial activity in *Drosophila* . Cell, 139(1), 149–160. 10.1016/j.cell.2009.07.034. 19804760 PMC2759400

[acel12876-bib-0175] Zuo, L. , He, F. , Tinsley, G. M. , Pannell, B. K. , Ward, E. , & Arciero, P. J. (2016). Comparison of high‐protein, intermittent fasting low‐calorie diet and heart healthy diet for vascular health of the obese. Frontiers in Physiology, 7, 350 10.3389/fphys.2016.00350. 27621707 PMC5002412

